# Predation risk increases in estuarine bivalves stressed by low salinity

**DOI:** 10.1007/s00227-021-03942-8

**Published:** 2021-07-24

**Authors:** Rula Domínguez, Elsa Vázquez, Isabel M. Smallegange, Sarah A. Woodin, David S. Wethey, Laura G. Peteiro, Celia Olabarria

**Affiliations:** 1 Centro de Investigación Mariña, Universidade de Vigo, Departamento de Ecoloxía e Bioloxía Animal, Facultade de Ciencias do Mar, 36331 Vigo, Spain; 2 Institute for Biodiversity and Ecosystem Dynamics, University of Amsterdam, Amsterdam, the Netherlands; 3 Department of Biological Sciences, University of South Carolina, 715 Sumter Street, Columbia, SC 29208 USA; 4 Instituto de Investigacións Mariñas Consejo Superior de Investigaciones Científicas, C/Eduardo Cabello, 6, 36208 Vigo, Spain

**Keywords:** Low salinity stress, Predator-prey interactions, Estuarine bivalves, Shore crab and gastropod, Shellfish bed

## Abstract

Salinity drops in estuaries after heavy rains are expected to increase in frequency and intensity over the next decades, with physiological and ecological consequences for the inhabitant organisms. It was investigated whether low salinity stress increases predation risk on three relevant commercial bivalves in Europe. In laboratory, juveniles of *Venerupis corrugata, Cerastoderma edule*, and the introduced *Ruditapes philippinarum* were subjected to low salinities (5, 10 and control 35) during two consecutive days and, afterwards, exposed to one of two common predators in the shellfish beds: the shore crab *Carcinus maenas* and the gastropod *Bolinus brandaris,* a non-indigenous species present in some Galician shellfish beds*.* Two types of choice experiment were done: one offering each predator one prey species previously exposed to one of the three salinities, and the other offering each predator the three prey species at the same time, previously exposed to one of the three salinities. Consumption of both predators and predatory behaviour of *C. maenas* (handling time, rejections, consumption rate) were measured. Predation rates and foraging behaviour differed, with *B. brandaris* being more generalist than *C. maenas*. Still, both predators consumed significantly more stressed (salinity 5 and 10) than non-stressed prey. The overall consumption of the native species *C. edule* and *V. corrugata* was greater than that of *R. philippinarum*, likely due to their vulnerability to low salinity and physical traits (e.g., thinner shell, valve gape). Increasing precipitations can alter salinity gradients in shellfish beds, and thus affect the population dynamics of harvested bivalves via predator–prey interactions.

**Supplementary Information:**

The online version contains supplementary material available at 10.1007/s00227-021-03942-8.

## Introduction

Salinity is an important factor in shaping the boundaries of species distributions, influencing small and large-scale biotic interactions (Berger and Kharazova 1997; Smyth and Elliott 2016). In estuaries, salinity values usually fluctuate. The nature of such fluctuations can be cyclic, due to the tidal regime, or episodic, due to increased river run-off after heavy rainfalls, evaporation in warmer conditions or anthropogenic inputs such as dam or industrial effluent discharges (Cardoso et al. 2008; Elliott and Whitfield 2011; Parada et al. 2012; Wolanksi and Elliott 2015). Organisms inhabiting estuarine areas have the ability to regulate their physiological, biological and behavioural responses to cyclic changes, while extreme salinity fluctuations may impair such responses (Verdelhos et al. 2015; Peteiro et al. 2018; Domínguez et al. 2020; Woodin et al. 2020) with far-reaching consequences for the survival and recruitment of these organisms (Beukema and Dekker 2005; Petes et al. 2007; Talmage and Gobler 2011; Vázquez et al. submitted).

Predator-prey interaction is one of the most important biotic processes in shaping the structure and dynamics of populations and communities of estuaries and other intertidal shores (Menge 1983; Wilson 1991; Rosa et al. 2008). Fluctuations in salinity may affect predator–prey dynamics in different ways (Seitz 2011; Smith et al. 2018). For instance, foraging efficiency may be altered since short periods of low salinity often limit predator activity, e.g., decreasing activity levels in starfish (Forcucci and Lawrence 1986), increasing searching time in gastropods (Zhang et al. 2017) and, in general, decreasing feeding rates (Garton and Stickle 1980; Stickle et al. 1985; Breen and Metaxas 2008). Alternatively, if the predator can avoid stress and the prey has limited movement, there can be a salinity threshold below which increased exposure becomes detrimental to prey resistance (Witman and Grange 1998; McLeod et al. 2008). Costs of maintenance of internal osmolality (Carregosa et al. 2014) may divert expenditures from other physiological and biological processes, such as feeding, respiration, growth, reproduction or anti-predator mechanisms (Kinne 1971; Akberali and Trueman 1985). Individual clams often respond to chemical cues released from a conspecific victim of predation or from a predator (Whitlow et al. 2003; Cheung et al. 2004; Smee and Weissburg 2006; Griffiths and Richardson 2006), and this response may be inhibited by stress.

The Galician shellfish beds (NW Spain) are a human-dominated environment located in estuarine areas called rias, which support important shellfisheries due to their high productivity (Figueiras et al. 2002). As a consequence, they suffer not only natural but also anthropogenic impacts (Alvarez et al. 2006; Filgueiras and Prego 2007; Parada et al. 2012; Villalba et al. 2014; Olabarria et al. 2016). Climate change is expected to directly affect the salinity patterns of estuaries in two major ways: (1) a continued rise in sea level, and (2) altered river flow via changes in pattern of rainfall and drought. Climate projections for the Atlantic European coast predict an increase in frequency and intensity of precipitation, primarily in the winter (Cardoso Pereira et al. 2020; Lorenzo and Alvarez 2020), as has already been observed in some areas (Cardoso et al. 2008; Grilo et al. 2011), but not in others (Sáez de Cámara et al. 2015).

The native venerid *Venerupis corrugata* (Gmelin, 1791), the introduced *Ruditapes philippinarum* (Adams and Reeve 1850), and the native cockle *Cerastoderma edule* (Linnaeus 1758) are three of the most relevant commercial species of bivalves in Europe, particularly in Galicia (NW Iberian Peninsula). The landings of these species represented an average of ~ 5.800 tonnes per year, the 71% of the total bivalve captures with an average market value of ~ 42 millions of euros per year in the period 2001–2020 (elaboration based on official data from www.pesca degalicia.com, last access May 2021). Because they occur in different habitats, i.e. position on the shore, burrowing ability, and also differ in morphological and behavioural traits (e.g. siphon length, shell thickness, valve gaping), these species differ in their abilities to cope with sudden salinity changes (Woodin et al. 2020; Domínguez et al. 2020) and in their resistance to predators (Whitlow et al. 2003; Curtis et al. 2012; Brom and Szopa 2016). Particularly *V. corrugata* is vulnerable to drops in salinity (Domínguez et al. 2020) as reflected in its higher densities in low intertidal and shallow subtidal habitats (Carregosa et al. 2014; Macho et al. 2016). All three species live in euryhaline conditions and are osmoconformers able to regulate to different extents their ionic concentrations to match the external environment, although their primary responses to salinity stress are behavioural, including valve closure (Shumway 1977; Akberali and Trueman 1985; Kim et al. 2001; Verdelhos et al. 2015; Domínguez et al. 2020) and burrowing (Woodin et al. 2020).

After a stressful event, or stressful period, bivalves need to resume filtration and excrete metabolic products of anaerobic metabolism to avoid toxicity (Griffiths and Griffiths 1987). Duration of valve closure following stress differs between species; for instance, fewer adults of *V. corrugata* and *C. edule* maintained closed valves compared to adults of *R. philippinarum* after salinities equal to and below 15 were applied (Domínguez et al. 2020). Below 15, burrowing activity of these species was also reduced (Woodin et al. 2020), which might increase the encounter rate between predator and prey. These traits can increase vulnerability to predation, not only facilitating the valve opening by the predator, but also the detection of prey by chemical cues (Hayden et al. 2007; Hay 2009).

Two species, the shore crab *Carcinus maenas* (Linnaeus 1758) and the gastropod *Bolinus brandaris* (Linnaeus 1758) are common predators of juvenile bivalves in the intertidal fishing beds (Seed 1993; Richards et al. 1999; Klassen and Locke 2007; Bañón et al. 2008; Smallegange et al. 2009; Dethier et al. 2019). The shore crab has been an invasive species for over a decade in a fishing bed in Ría de Arousa (42° 29′25″N, 8° 50′24″W, Bañón et al. 2008). It can cope with low salinities by efficient osmoregulation of its extracellular fluid (Jillette et al. 2011; Klassen and Locke 2007), maintaining activity (Breen and Metaxas 2008; Curtis et al. 2012) or avoiding short-term stress by an increased locomotor activity defined as halokinesis (Thomas et al. 1981; McGaw et al. 1999). The impact of low salinity on *B. brandaris* is less studied; Dalla Via and Tappeiner (1981) found lower activity and oxygen consumption under low salinities. Together, these two predators can have a strong impact on the abundance, composition and dynamics of bivalve populations in shellfish beds. Their feeding strategies differ due to their morphologies. Shore crabs use their claws (chelipeds) to probe into the sediment and find prey and, they can exhibit two distinctive feeding techniques. When they feed on small prey, the minor chela immobilizes the prey and the major chela crushes the shell. When they are forced to feed on larger prey, with risk of claw damage, the crabs adopt a slower technique of cutting along the valve’s edges (Smallegange and van der Meer 2003). They can dig down some centimetres in the sediment, causing an escape response in bivalves by burrowing deeper (Whitlow et al. 2003). The feeding strategy of *B. brandaris* is less studied and may be similar to that or other muricids. The smaller individuals drill prey shell, whereas marginal chipping is adopted more frequently by larger gastropods (Peharda and Morton 2006). They can also adopt different techniques depending on bivalve shell thickness. It was found that *Tapes spp.* were consumed by chipping the shell margin, while thinner shelled bivalves were accessed using the labral spine in the border of the operculum aperture to push prey’s valves, breaking them followed by proboscis insertion (Morton et al. 2007). However, the labral spine is a feature that varies upon species (Marko and Vermeij 1999) and seems to be absent in *B. brandaris* (personal obs.). This species may likely use the inner lip of their shell to open the bivalve and suck the glandular tissue of the clam with its proboscis.

Predator consumption is a combination of both predator and prey traits (e.g., prey size, prey vulnerability, prey availability, quality or profitability, handling time, or the past experience of the predator and prey) (Munari and Mistri 2011). Here, we carried out laboratory experiments to investigate the predation activity and consumption rates of two predator species, *C. maenas* and *B. brandaris*, on juvenile bivalves previously exposed to short-term salinity stress levels that reflect those occurring in Galician shellfish beds. The hypotheses tested were that (1) predation rates would be greater on the more stressed individuals (exposed to salinities of 5 or 10 > exposed to 35), and (2) predators would consume more vulnerable species, depending on their shell morphology, burrowing capacity and valve closure ability (*C. edule* > *V. corrugata* > *R. philippinarum*). With regard to predator behaviour, the hypotheses tested were that (1) handling time and (2) rejection rate would be highest for the least stressed prey, and for the species with shells most resistant to crushing, *R. philippinarum* > *C. edule* > *V. corrugata* (Coffen-Smout 1998).

## Materials and methods

The experiments were performed at Estación de Ciencias Mariñas de Toralla (CIM-ECIMAT; www.cim.uvigo.gal) of Universidade de Vigo (NW Spain), in May 2017.

### Animal collection and maintenance

Individuals of *V. corrugata* and *R. philippinarum* were provided by hatcheries in Galicia, whereas juvenile cockles *C. edule* were collected at Ría de Noia (42° 47′ 0″ N, 8° 53′ 0″ W) and transported to the laboratory in refrigerated boxes. Clams were kept for several days (minimum 1 week, maximum 4 weeks) in baskets hanging off the ECIMAT dock to acclimatize to natural conditions. On the day prior to salinity stress, bivalves were taken to the laboratory and measured to the nearest 1 mm. Bivalves of sizes 19–24 mm in maximum shell length were selected and placed in a total of 64 or 60 (depending on the experiment) 1 L-plastic beakers (17 cm tall, 10.8 cm diameter) filled with sand from a nearby intertidal area for use in the experiments. Sand was extracted from approximately the upper 4 cm of sediment at Canido (42° 11′ 36.27″ N 8° 47′ 50.15″ W), a semi-protected intertidal sand flat with mean grain size of 0.19 mm (see Woodin et al. 2020). Such sizes are those that are normally seeded into the shellfish beds. Size at first sexual maturity of *R. philippinarum* is 29.4 mm in shell length (i.e., before 1 year old) (Moura et al. 2017), of *V. corrugata* is 22 mm (Maia et al. 2006), and of *C. edule* is 15–19 mm (Mejuto 1984; Pérez-Camacho and Román 1984). Therefore, throughout this paper, the term juvenile was maintained for all prey, although some individuals of *C. edule* and *V. corrugata* could fall out of this category.

The predators, *C. maenas* and *B. brandaris*, were manually collected with the collaboration of the fisher’s guilds from the shellfish beds of Noia (42° 47′0″N, 8° 53′0″W) and O Grove (42° 49′6″N, 8° 86′5″W) and transported to the laboratory in refrigerated boxes. Size range of *C. maenas* was from 28 to 58 mm carapace width (45.7 ± 6.5; mean ± S.D) and of *B. brandaris* from 61 to 87 mm shell length (69.4 ± 5.2). Once in the laboratory, they were maintained in PVC flow-through tanks with 50 μm-filtered seawater placed in separate rooms. Tanks with shore crabs and gastropods were provided with rocks and a fine bottom-layer of sand (⁓1 cm depth), respectively, to mimic natural conditions in the field. A maximum of 20 shore crabs and 12 gastropods were kept in each 110 L (*n* = 6, dimensions 80 × 60 × 33) and 80 L (*n* = 6, dimensions 80 × 60 × 22 cm) tanks, respectively, in rooms with ambient temperature at ~ 18–20 ºC and a 16:8 h light: dark photoperiod. Seawater temperature in tanks was 19.1 ± 0.6 ºC corresponding to average sea surface temperature recorded in the Ria de Vigo at that time of the year (www.meteogalicia.gal). Predators were fed daily with fresh clams of the same species as offered in the experiment. Feeding was ceased, and all food remains were removed from the tanks 24 h before the start of the experiment in case of *C. maenas,* and 48 h before the start of the experiment in case of *B. brandaris*. A total of 2790 bivalves and 186 predators were used in the experiments.

### Experiment setup

Prior to the predation experiments, prey bivalves were exposed to salinity stress treatments for a period of 48 h. Predators were not exposed to salinity stress treatments.

#### Prey salinity stress

**Fig. 1:**
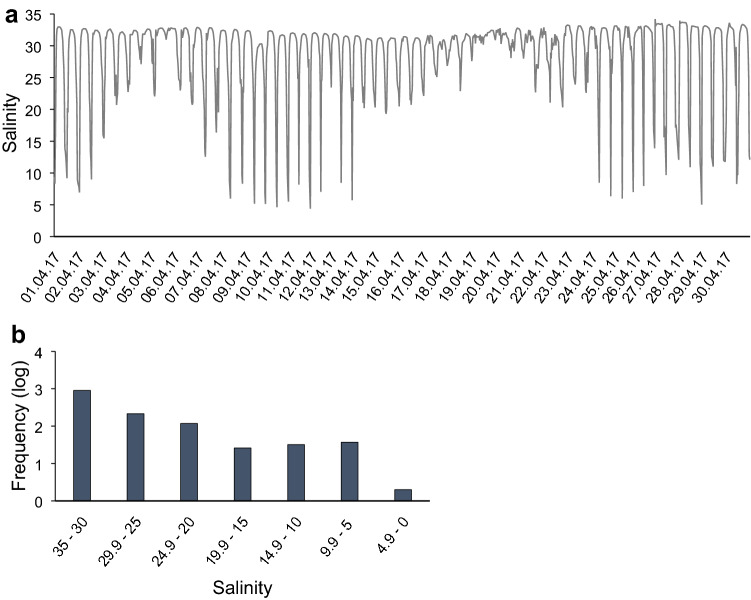
Salinity data for a shellfish bed in Cambados, Ría de Arousa during April 2017 registered with miniCTDs placed in the first 5 cm of the water column, near the sediment: **a** salinity profile, and, **b** frequency (log scale) of each salinity range

Salinity treatments were defined according to our field records taken over the previous years (2015–2017) by Mini-CTDs (Star Oddi^®^) placed in the first bottom centimetres of the water column at local shellfish beds. These records indicated steep salinity drops during low tides (Fig. 1) with values as low as 5 for longer periods after heavy rains, as reported previously by Parada et al. (2012). A clear physiological salinity threshold at 15 for the studied species was detected by Domínguez et al. (2020). Taking this into account, the experimental salinity treatments were set at 5 and 10 for the stress, and 35 for the control treatment (S5, S10 and S35, hereinafter). The stress was maintained continuously for the duration of the 2 days; longer exposure would lead to mortality of *C. edule* (Verdelhos et al. 2015).

To expose bivalves to salinity treatments, three 480 L tanks (S5, S10 and S35) filled with 50 μm-filtered seawater and constant aeration were setup in a room at ambient temperature, ~ 18–20 °C. The salinity drop was simulated by mixing 50 μm-filtered seawater with dechlorinated freshwater to reach the treatment salinity in each of the three tanks. Salinity was measured by salinity probes (Hach, HQ40d) positioned at the bottom and top of the tanks. This salinity treatment was maintained for 2 consecutive days, with aeration and periodic correction of salinity if necessary, by adding dechlorinated freshwater, as slight increases happened due to evaporation. In any case, such effects can be similar to those experienced in the field. Five individuals of the same species were placed at the sediment surface of each beaker, which had 2 bottom orifices of 1 cm diameter covered by 80 μm mesh to avoid sediment loss, but to allow a water flux through the column of sediment. Bivalves were allowed to burrow, and those that did not burrow within 8 h, were replaced. Following the burrowing period the beakers with the bivalves were placed in the low salinity tanks for 48 h. The bivalves were offered food every day (microalgae mixture of *Isochrysis galbana*, *Tetraselmis suecica*, *Chaetoceros gracilis* and *Rhodomonas lens*, constituting a 1% maintenance diet based on a dry weight of 0.68 g, same procedure as in Domínguez et al. 2020). For each experimental salinity, a total of 16 plastic beakers were submerged in each of the three tanks.

#### Predation (“cafeteria”) experiments

**Fig. 2:**
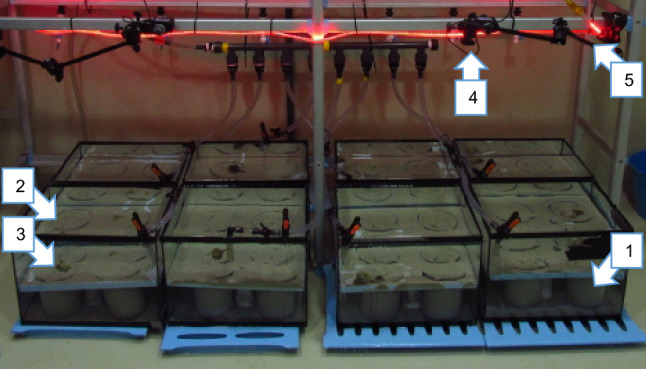
Setup of the mesocosm experiment: 1) aquarium, 2) container with sand and bivalves, 3) predator, 4) camera, 5) red led light

After the stress period, the plastic beakers filled with sediment and with the bivalves were immediately transferred to aquaria with running seawater at ambient salinity, ~ 35. Aquaria were placed in an experimental room with controlled air temperature (18–20 ºC) and a 16:8 h light: dark photoperiod, with red lights while photo recording at night to minimize disturbing animals. In each aquarium, four plastic beakers were fully inserted in a polystyrene platform (50 × 40 cm of surface, approximately) with custom-made holes of the size of the containers; then, the platform and upper part of the beakers were completely covered by sand (Fig. 2). All plastic beakers were randomly located within the aquarium, and aquaria were randomly located within the experimental room. Four synchronized photo cameras, each located above each group of four experimental aquaria, took one photograph every 30 s, over a period of 24 or 48 h, to record prey and predator behaviour. Preliminary recording indicated that crabs rarely altered behaviours such as manipulation of a bivalve, within 30 s.

**Fig. 3:**
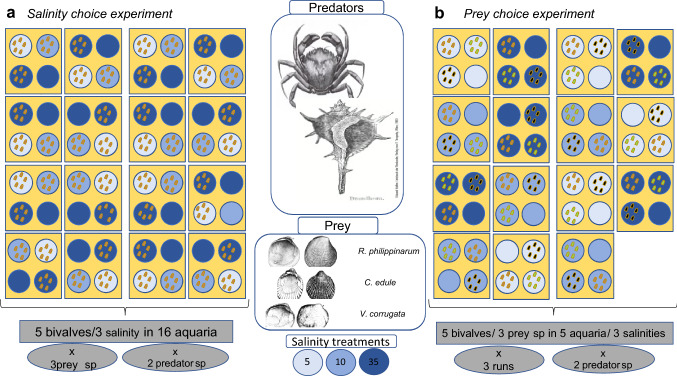
Design of the mesocosm experiment. Each aquarium with four small beakers inside filled with sediment to allow bivalves to burrow. In each beaker, 5 juvenile bivalves were seeded in the *Salinity choice experiment* **a**: aquaria with bivalves of the same species stressed at different salinity treatments and, *Prey choice experiment* **b**: aquaria with 5 bivalves of each prey species under the same salinity treatment; sp is abbreviation for species

Two different choice experiments were consecutively done to further investigate predation on the same prey species, but with different levels of salinity stress, designated as *salinity choice experiment* (Fig. 3a), and the predation upon the three species with the same level of stress, called *prey choice experiment* (Fig. 3b).

For the *Salinity choice experiment,* three beakers with the same species of bivalves previously exposed to each of the salinity treatments (S5, S10, S35), and an additional procedural control beaker without bivalves only with sand and kept at S35, were randomly placed in each of the 16 aquaria. The procedural control was a choice alternative for the predator. One predator was introduced in the middle of each aquarium, and then the aquarium was covered with a mesh to prevent escape. Predators were left for 24 h and 48 h in case of *C. maenas* and *B. brandaris*, respectively, as the gastropod has lower predation rates (pers. obs.). At the end of each run, the numbers of prey eaten (empty shells), dead (valves opened with flesh inside) or alive (either buried or unburied) were recorded. Predators, bivalves and sediment were not reused in subsequent experiments.

For the *Prey choice experiment*, three beakers, each one with a different species stressed at the same salinity treatment, and an additional procedural control beaker with only sand, were randomly placed in each of the 15 aquaria; 5 aquaria per each salinity treatment. This procedure was repeated over the course of three consecutive days to increase the number of replicates (5 aquaria × 3 runs, *n* = 15 beakers of each combination of treatment and species). Predators were introduced following the same procedure as the previous experiment. Since aquaria had to be reused for consecutive runs, all elements were removed and cleaned with fresh water in between runs and the sediment replaced with new sediment. Predators, bivalves and sediment were not reused in subsequent experiments.

### Response variables

To investigate the effect of salinity stress on predation rates, prey selection and predation behaviour, different response variables were measured and calculated.

(i) Total prey consumption (number of prey consumed during a run: 24 h for *C. maenas* and 48 h for *B. brandaris*).
(ii) Early prey consumption (number of prey consumed in the first 4 h) for *C. maenas*.
(iii) Handling time to first bite (HBT) (number of min of physical manipulation of a bivalve including excavating it from the sediment, ending in a first bite, measured during the first 4 h of each run) for *C. maenas*.
(iv) Handling time plus period of consumption (HCT) (number of min of physical manipulation of a bivalve for its opening and then consumption until the crab abandoned the clam, measured during the first 4 h of each run) for *C. maenas*.
(v) Burrowing time of *C. maenas* (burrowing within the sediment in a beaker, during the first 4 h of each run).
(vi) Total rejections (number of times a predator rejected a handled prey, during the first 4 h of each run) for *C. maenas*.
(vii) Fraction consumed upon capture by *C. maenas*, calculated as number of consumptions/(number of rejections+number of consumptions).

Handling time was calculated assuming that behaviour was the same during the 30 s interval between the two sequential photographs. Two metrics are given, first the combined handling time plus consumption time measurement (HCT) and second handling time to first bite (HBT). Crab behaviour once the bivalve was opened, i.e. the end of handling time, was to take a bite and then move slightly away while macerating the bite. This is HBT and is likely very close to true handling time, which we could not determine in ~ 20 percent of the cases given crab positioning and camera angles. All crab handling data are reported conservatively as HBT. We did not analyse the foraging behaviour of the gastropod *B. brandaris* because it was not possible to observe its behaviour from zenithal photographs.

Additionally, information on the number of bivalves found on the sediment surface at the beginning of each run and the position and state of the bivalves remaining at the end was recorded. These data were not analysed statistically because there were a number of inconsistencies between data recorded at the end of the experiments and the real position of the bivalves registered on the pictures, due to predator activity moving bivalves between beakers. They served, however, to show the main trends on prey availability.

### Statistical analysis

Aquaria from which predators escaped, were inactive, or spent more than half of the analysed period buried, were discarded from the analyses. The final number of replicates of each analysis is indicated in the tables below.

To test the effect of salinity on total and early consumption upon each prey species, and rejections, Generalized Linear Mixed effects models (GLMMs) were used, with a Poisson error distribution. Fraction consumed was analysed using a Linear Mixed Model (LMM) and handling time (HBT and HCT) using GLMMs, with a negative binomial distributions of errors. In the *Salinity choice experiment* models considered Salinity as fixed factor, Size as covariate, and Aquarium as random term, whereas in the *Prey choice experiment*, the fixed factor considered was prey Species. In the *Salinity choice experiments* the individual crabs could choose among clams of one species previously exposed to different salinities, so the fixed factor was Salinity. In the *Prey choice experiments* the individual crabs could choose among clams of different species, all of whom had been exposed to a single salinity so the fixed factor was prey Species. Size of the predator was standardized by subtraction of the mean, and was included as a covariate to control for any effects caused by the size differences between individuals (Zuur et al. 2009). Because male and female crabs were not evenly distributed across treatments, we could not test for the effect of sex, and excluded it from the models. We find this justified because sex may not be a key parameter in the shore crab’s ability to consume clams, similar to the assumptions of Dethier et al. (2019), and our preliminary analysis confirmed this. Besides, the size of appendices may determine the selection of prey size (Elner 1980) and it is correlated with carapace width, which was measured and analysed.

We tested for homogeneity among slopes of the main treatments by including the interaction term ‘covariate × main factor’ in the model. With a non-significant interaction term, homogeneity of slopes was assumed and the model excluding the interaction was re-run (McDonald 2009). The same analysis was applied to test the effect of salinity and prey species on consumption in the *Prey choice experiment,* and, in this case, factor Run and Aquarium were both considered as random terms.

The assumptions of normality and homogeneity of variances were checked by visual inspection of Q-Q plots and Levene’s test and Fligner-Killeen’s test, respectively. These tests resulted significant only for the total number of eaten *C. edule* and early eaten in the first 4 h in the *Salinity stress experiment*. However, as the *p* value was close to 0.05 data were left untransformed, except for fraction consumed that was arcsine-square root transformed. Overdispersion was also assessed for all models. Analyses were done using the *lme4* package (Bates et al. 2015) and *lmerTest* package (Kuznetsova et al. 2017); *car* package was used for testing the significance of the models by Anova (Fox and Weisberg 2019) in R version 3.6.1 (R Core Team 2019). Post-hoc tests were performed using *emmeans* package (Lenth 2020).

## Results

### Experiments with the predator *C. maenas*

#### Salinity choice experiment

**Fig. 4:**
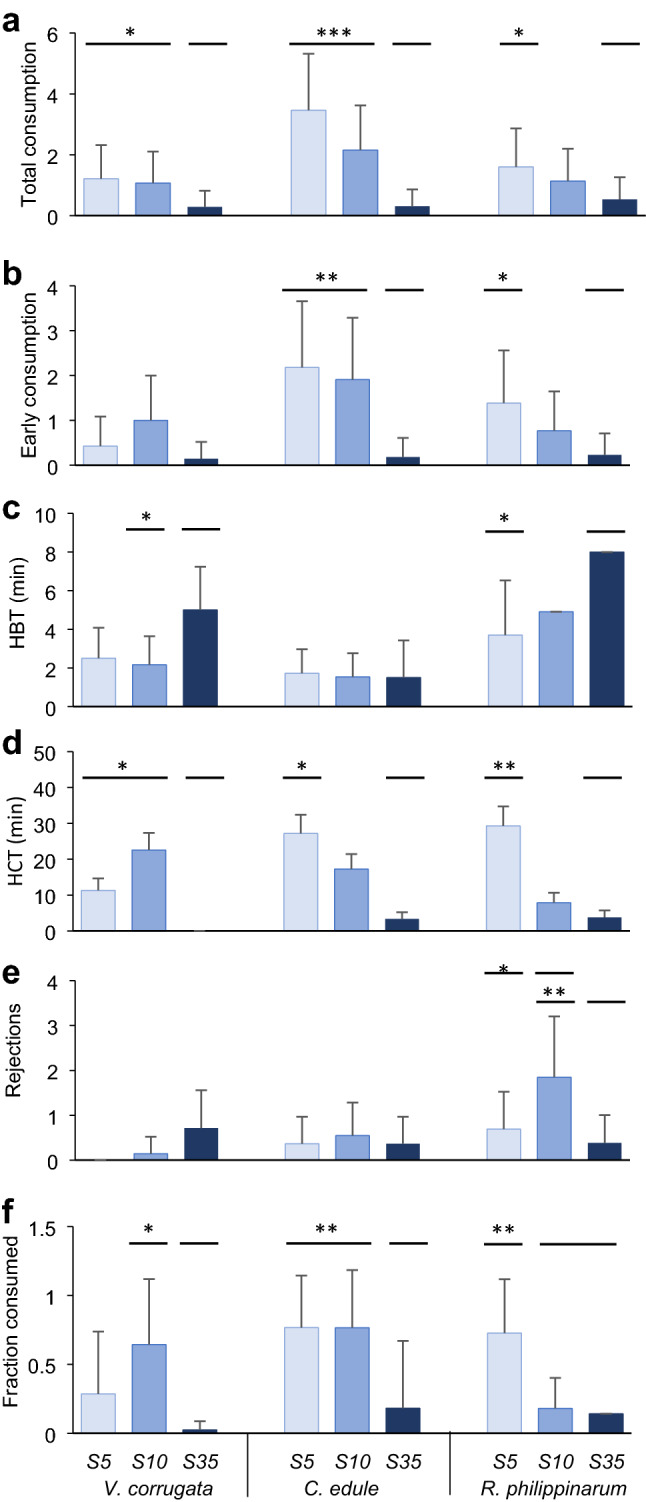
Results of the *Salinity choice experiment* with *C. maenas.* Mean (+ SD, *n* = 8–15) of **a** total prey consumption in 24 h, **b** number of prey consumed in the first 4 h, **c** HBT: handling time to the first bite in the first 4 h, **d** HCT: handling time plus consumption time in the first 4 h, **e** number of rejections in the first 4 h, and f) fraction consumed upon capture in the first 4 h by treatment (S5, S10, S35) for each prey species (*V. corrugata*, *C. edule, R. philippinarum).* Horizontal bars indicate significant differences between levels of the factor treatment, found by the post-hoc tests, *p < 0.05, **p < 0.01, ***p < 0.001. Marginal differences not shown

In the *Salinity choice experiment*, where the prey species was the same but with different levels of salinity stress, non-stressed prey were consumed significantly less by the predator (Fig. 4, Table 1). The total number of *V. corrugata* consumed by *C. maenas* was significantly larger in S5 and S10 than in S35, as was also true for *C. edule* (Fig. 4a, Table 1). In the case of *R. philippinarum*, the trend was the same although only individuals in S5 suffered significantly greater predation compared to S35 (Fig. 4a).

**Table 1:** Summary of GLMM testing the effect of salinity treatments on total consumption of prey in 24 h for the *Salinity choice experiment* with *C. maenas*

Variable	Prey Species	Replicates (Aquarium)	Full Model	Parameter	*χ*^2^	df	*p*	Variance	SD	Post-hoc	*p*
Total consumption	*V. corrugata*	*n* = 14	Cons ~ Treat + Size + (1|Aquarium)	Random effect							
				Aquarium				0.08	0.29		
				Fixed effects							
				Intercept	0.11	1	0.736				
				Treat	7.02	2	**0.029**			S5-S35	0.024
				Size	3.21	1	**0.073**			S10-S35	0.048
	*C. edule*	*n* = 13	Cons ~ Treat + Size + (1|Aquarium)	Random effect							
				Aquarium				0.006	0.08		
				Fixed effects							
				Intercept	66.88	1	**2.8e-16**				
				Treat	22.88	2	**1.1e-05**			S5-S35	< 0.001
				Size	0.76	1	0.383			S10-S35	< 0.001
	*R. philippinarum*	*n* = 15	Cons ~ Treat + Size + (1|Aquarium)	Random effect							
				Aquarium				0	0		
				Fixed effects							
				Intercept	4.67	1	**0.031**				
				Treat	7.29	2	**0.026**			S5-S35	0.019
				Size	2.05	1	0.151				

The model used Salinity treatment (Treat) as fixed factor, Aquarium as random factor and predator size as covariate. Post-hoc tests results for *Salinity treatment* (S5, S10, S35) are shown. Data were not transformed

Values in bold are statistically significant (*p* < 0.05)

Mean early prey consumption followed the same pattern, with the exception of *V. corrugata*, which was similarly little consumed in all treatments (Fig. 4b, Table S1)*.*

The handling time to the first bite (HBT) differed significantly among treatments for *V. corrugata* (*χ*^2^: 6.12, df 2, *p* = 0.04), due to higher values in S35 compared to S10, and *R. philippinarum* (*χ*^2^: 5.55, df 2, *p* = 0.06), due to higher values in S35 compared to S5 (Fig. 4c, Table S1).

The composite of handling and consumption time (HCT) differed significantly among treatments for all prey species (Fig. 4d, Table S1). For *V. corrugata,* differences in HCT (*χ*^2^: 49.62, df 2, *p* < 0.001) were due to higher values in S5 and, particularly, in S10 compared to S35*.* In the case of *C. edule*, significant differences among treatments for HCT (*χ*^2^: 7.85, df 2, *p* = 0.02) were related to longer times in S5, and marginally in S10 (*p* = 0.051 and 0.09, respectively), than S35. For *R. philippinarum*, differences among treatments for HCT (*χ*^2^: 17.02, df 2, *p* < 0.001) were due to significantly higher values in S5 compared to S35, and marginally significant compared to S10 (*p* = 0.09).

The number of *R. philipinarum* individuals rejected by *C. maenas* differed significantly among treatments (*χ*^2^:15.81, df 2, *p* < 0.001), with more rejections in S10 than S5 and S35. In contrast, the number of *V. corrugata* and *C. edule* individuals rejected was small, i.e., on average less than 1 (Fig. 4e, Table S1). Variability among aquaria was large for all prey species as shown by the large SD values and the variance explained by the random factor (Table S1).

*Carcinus maenas* consumed a greater fraction of the most stressed prey than of the clams from the other treatments (Fig. 4f, Table S1). The fraction consumed by *C. maenas* differed significantly among treatments for all prey species (*V. corrugata*: *χ*^2^: 9.92, df 2, *p* < 0.01; *C. edule*: *χ*^2^:15.55, df 2, *p* < 0.001; *R. philippinarum*: *χ*^2^:34.32, df 2, *p* < 0.001, Fig. 4f, Table S1). The fraction of *V. corrugata* consumed was significantly greater in S10 than S35. In the case of *C. edule*, it was significantly greater in S5 and S10 than in S35, whereas in *R. philippinarum*, consumption rate was significantly greater in S5 than S10 and S35.

#### Prey choice experiment

**Fig. 5:**
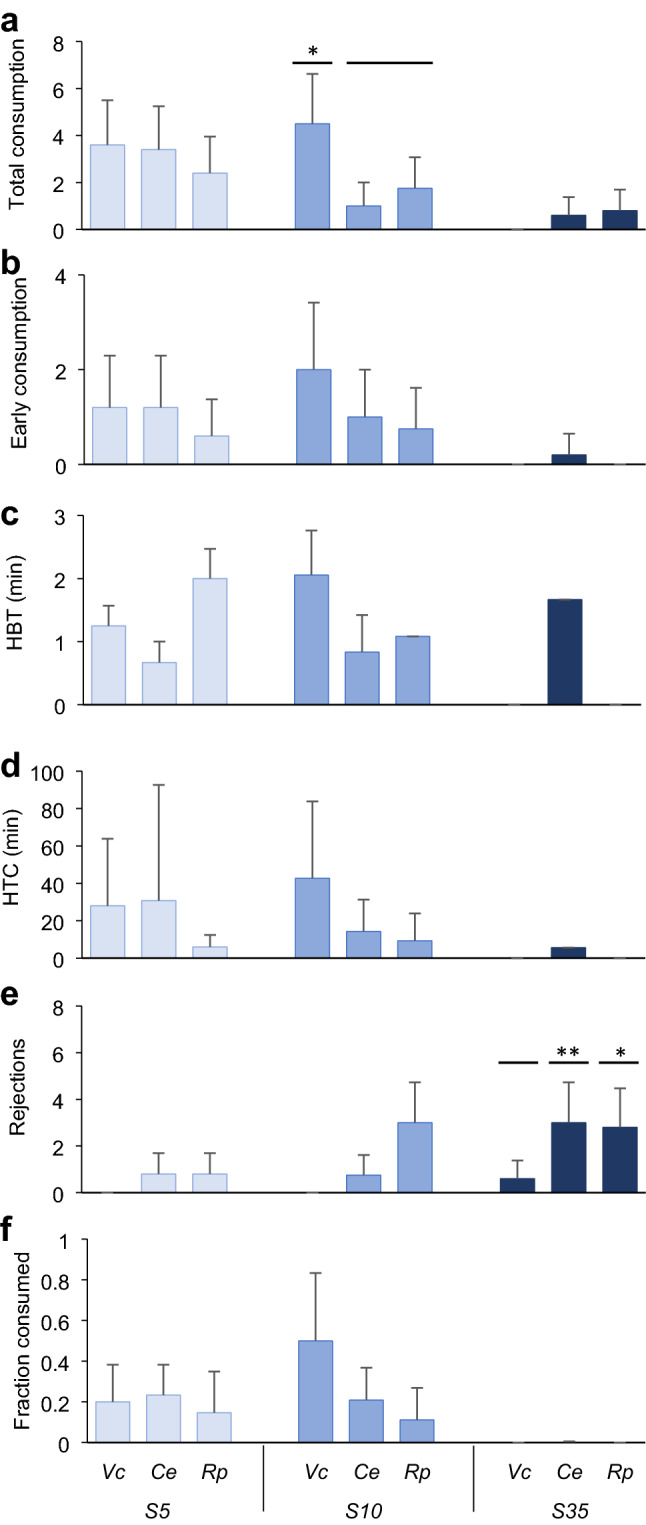
Results of the *Prey choice experiment* with *C. maenas*. Mean (+ SD; *n* = 18–45) of **a** number of eaten prey in 24 h, **b** number of eaten prey in the first 4 h, **c** HBT: handling time to the first bite in the first 4 h, **d** HCT: handling time plus consumption time in the first 4 h, **e** number of rejections in the first 4 h, and **f** fraction consumed upon capture in the first 4 h by treatment (S5, S10, S35) and prey species (*Vc*: *V. corrugata*, *Ce*: *C. edule, Rp*: *R. philippinarum)*. Horizontal bars indicate significant differences between levels of the factor treatment, found by the post-hoc tests, *p < 0.05, **p < 0.01, ***p < 0.001. Marginal differences not shown

When prey species and treatments were considered together in the *Prey choice experiment*, the general trend was that total consumption of predators was greater on stressed prey, mainly on stressed *V. corrugata* than on the other species (Fig. 5a). Post-hoc tests found differences among species in S10, with the greatest total consumption upon *V. corrugata* compared to the other two species (Table 2). Consumption upon non-stressed prey was smaller compared to the stressed individuals of the three species.

**Table 2:** Summary of GLMM testing the effect of salinity treatments (S5, S10, S35) on total consumption of prey in 24 h for the *Prey choice experiment* with *C. maenas*

Variable	Salinity treatment	Replicates (Aquarium)	Full model	Parameter	*χ*^2^	df	*p*	Variance	SD	Post-hoc	*p*
Total consumption	S5	*n* = 42	Cons ~ Species + Size + (1|Aquarium) + (1|Run)	Random effect							
				Aquarium				0	0		
				Run				0.08	0.29		
				Fixed effects							
				Intercept	0.56	1	0.452				
				Species	1.31	2	0.521				
				Size	0.02	1	0.877				
	S10	*n* = 45	Cons ~ Species + Size + (1|Aquarium) + (1|Run)	Random effect							
				Aquarium				0	0		
				Run				0.01	0.11		
				Fixed effects							
				Intercept	5.89	1	**0.015**				
				Species	11.01	2	**0.004**			Vc-Ce	0.014
				Size	0.15	1	0.696			Vc-Rp	0.038
	S35	*n* = 45	Cons ~ Species + Size + (1|Aquarium) + (1|Run)	Random effect							
				Aquarium				< 0.01	< 0.01		
				Run				< 0.001	< 0.001		
				Fixed effects							
				Intercept	0	1	0.998				
				Species	0.14	2	0.931				
				Size	2.22	1	0.136				

The model used prey Species as fixed factor, Aquarium and Run as random factors and predator size as covariate. Post-hoc tests results for *Species* (Vc *V. corrugata*, Ce *C. edule*, Rp* R. philippinarum*) are shown. Data were not transformed

Values in bold are statistically significant (*p* < 0.05)

The early consumption in the *Prey choice experiment* differed among prey species in S10 (*χ*^2^: 7.54, df 2, *p* = 0.023), with a trend towards a greater consumption on *V. corrugata* than on *R. philippinarum* (*p* = 0.068). Almost no early consumption on non-stressed prey was found (Fig. 5b, Table S2).

The handling time to the first bite (HBT) and handling and consumption time (HCT) did not significantly differ among prey species in any treatment, except in S10 for HBT (*χ*^2^: 6.25, df 2, *p* = 0.044), due to a longer handling time of crabs before starting to eat *V. corrugata* compared to the other two species, although such trend was not detected by post-hoc tests (Fig. 5c, d, Table S2). Crabs tended to spend less time handling the most stressed *V. corrugata* and *C. edule* before starting to eat them (Fig. 5c) and more time to consume them completely (Fig. 5d).

The number of prey rejected by predators differed significantly in S35 (*χ*^2^: 7.71, df 2, *p* = 0.021, Table S2) and the crab size (*χ*^2^: 5.21, df 1, *p* = 0.022, Table S2), due to a greater rejection of *C. edule* and *R. philippinarum* compared to *V. corrugata* (*p* = 0.01, 0.04, respectively, Fig. 5e, Table S2). Also, larger crabs rejected a smaller number of prey in S35 (data not shown). For this variable, the random factors contributed considerably to explain part of the variance (i.e. the large variance explained by run and aquaria, Table S2).

The fraction of clams consumed by *C. maenas* differed significantly among species in S10 (*χ*^2^: 5.64, df 2, *p* = 0.059, Table S2, Fig. 5f), although the post-hoc tests were only marginally significant with the greatest fraction consumed for *V. corrugata* compared to *R. philippinarum* (*p* = 0.073, Table S2). The lowest values for all species were found in S35.

### Experiments with the predator *B. brandaris*

#### Salinity choice experiment

**Fig. 6:**
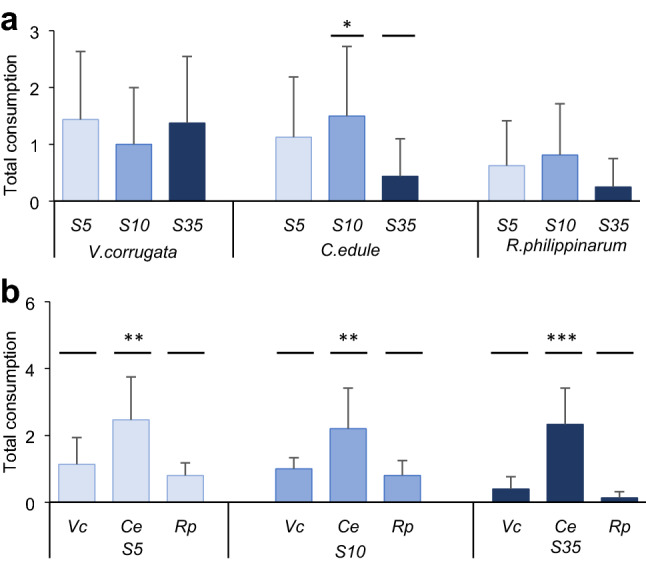
Results of the experiments with *B. brandaris.* Mean (+ SD, *n* = 16–45) of **a** number of eaten prey in 48 h by treatment (S5, S10, S35) and prey species (*V. corrugata*, *C. edule, R. philippinarum)* in the *Salinity choice experiment*, **b** number of eaten prey in 48 h by treatment (S5, S10, S35) and prey species (*Vc*: *V. corrugata*, *Ce*: *C. edule, Rp*: *R. philippinarum)* in the *Prey choice experiment*. Horizontal bars indicate significant differences among factors, found by the post-hoc tests, *p < 0.05, **p < 0.01, ***p < 0.001. Marginal differences not shown

In the *Salinity choice experiment* (Fig. 6a, Table 3), the consumption of *B. brandaris* was greater on stressed than non-stressed *C. edule*. The consumption of *V. corrugata* and *R. philippinarum* was not significant relative to the salinity treatment, although a trend towards a greater consumption of stressed than non-stressed *R. philippinarum* was observed (Fig. 6a). There was a significant effect of the predator’s size on the consumption on *V. corrugata*, with smaller *B. brandaris* showing greater consumption (Table 3) and a similar trend for *C. edule*.

**Table 3:** Summary of GLMM testing the effect of salinity treatments on total consumption of prey in 48 h for the *Salinity choice experiment* with *B. brandaris* as predator

Variable	Prey Species	Replicates (Aquarium)	Full model	Parameter	*χ*^2^	df	*p*	Variance	SD	Post- hoc	*p*
Total consumption	*V. corrugata*	*n* = 16	Cons ~ Treat + Size + (1|Aquarium) + (1|Run)	Random effects							
				Aquarium				0.09	0.29		
				Fixed effects							
				Intercept	5.84	1	0.016				
				Treat	1.39	2	0.497				
				Size	4.98	1	**0.026**				
	*C. edule*	*n* = 16	Cons ~ Treat + Size + (1|Aquarium) + (1|Run)	Random effects							
				Aquarium				0	0		
				Fixed effects							
				Intercept	2.71	1	0.099			S5-S35	0.085
				Treat	8.23	2	**0.016**			S10-S35	**0.011**
				Size	2.92	1	0.087				
	*R. philippinarum*	*n* = 16	Cons ~ Treat + Size + (1|Aquarium) + (1|Run)	Random effects							
				Aquarium				1e–14	1e–7		
				Fixed effects							
				Intercept	1.27	1	0.259				
				Treat	3.67	2	0.159				
				Size	0.89	1	0.344				

The models used Salinity treatment (Treat) as fixed factor, Aquarium and Run as random factors and predator size as covariate. *Pos hoc* tests results for the significant factor Treatment (S5, S10, S35) are shown. Data were not transformed

Values in bold are statistically significant (*p* < 0.05)

#### Prey choice experiment

In the *Prey choice experiment*, consumption differed significantly among species. *Bolinus brandaris* preyed more intensely on *C. edule* compared to *V. corrugata* and *R. philippinarum* in each salinity treatment (Fig. 6b, Table 4). Consumption by predators tended to be greater on stressed than on non-stressed *V. corrugata* and *R. philippinarum*.

**Table 4:** Summary of GLMM testing the effect of salinity treatments (S5, S10, S35) on total consumption of prey in 48 h for the *Prey choice experiment* both with *B. brandaris* as predator

Variable	Salinity treatment	Replicates (Aquarium)	Full model	Parameter	*χ*^2^	df	*p*	Variance	SD	Post-hoc	*p*
Total consumption	S5	*n* = 45	Cons ~ Sp * Size + (1|Aquarium) + (1|Run)	Random effects							
				Aquarium				< 0.01	0.05		
				Run				0	0		
				Fixed effects							
				Intercept	3.03	1	0.082				
				Sp	6.97	2	**0.031**			Vc-Ce	**0.015**
				Size	3.36	1	**0.067**			Ce-Rp	** < 0.001**
				Sp:Size	6.61	2	**0.038**				
	S10	*n* = 45	Cons ~ Sp + Size	Random effects							
			+ (1|Aquarium) + (1|Run)	Aquarium				0	0		
				Run				0	0		
				Fixed effects							
				Intercept	0.66	1	0.416				
				Sp	12.03	2	**0.002**			Vc-Ce	**0.03**
				Size	0.67	1	0.412			Ce-Rp	** < 0.01**
	S35	*n* = 45	Cons ~ Sp + Size	Random effects							
			+ (1|Aquarium) + (1|Run)	Aquarium				0	0		
				Run				0	0		
				Fixed effects							
				Intercept	0.06	1	0.79				
				Sp	28.86	2	** < 0.001**			Vc-Ce	** < 0.001**
				Size	< 0.01	1	0.923			Ce-Rp	** < 0.001**

The models used prey Species (Sp) as fixed factors, Aquarium and Run as random factors and predator size as covariate. *Pos hoc* tests results for the significant factor Species (Vc *V. corrugata*, Ce *C. edule*, Rp *R. philippinarum*) are shown. Data were not transformed

Values in bold are statistically significant (*p* < 0.05)

### Position in the sediment and state of prey in the experiments

**Fig. 7:**
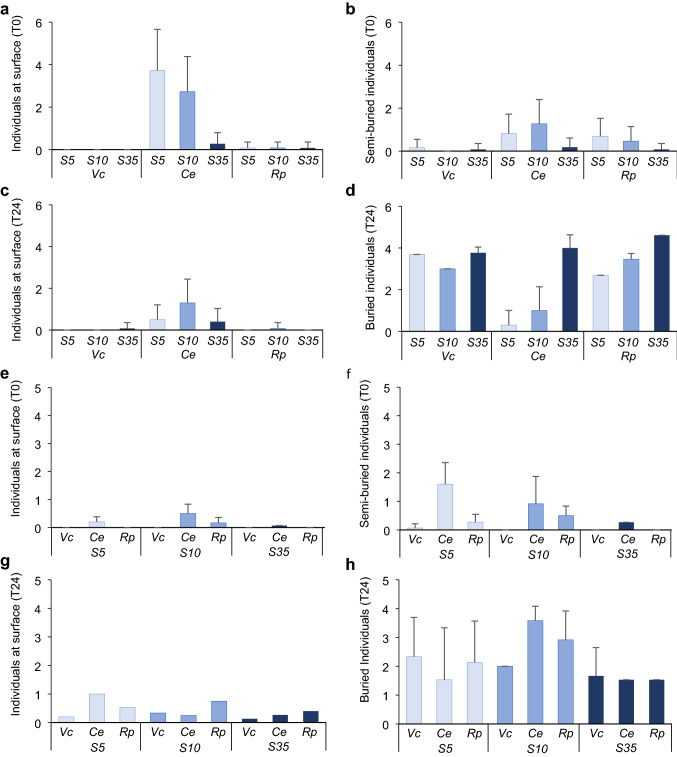
Results of the experiments with *C.maenas.* Mean (+ SD, *n* = 16–45) number of **a** individual bivalves found at surface and **b** semi-buried individuals at the beginning of the experiment (T0), **c** individuals found at surface and **d** buried individuals at the end of the experiment (T24) by treatment (S5, S10, S35) and prey species (*Vc*: *V. corrugata*, *Ce*: *C. edule, Rp*: *R. philippinarum*) in the *Salinity choice experiment,* **e** individual bivalves found at surface and **f** semi-buried individuals at the beginning of the experiment (T0), **g** individuals found at surface and **h** buried at the end of the experiment (T24) by treatment (S5, S10, S35) and prey species (*Vc*: *V. corrugata*, *Ce*: *C. edule, Rp*: *R. philippinarum*) in the *Prey choice experiment*

When using *C. maenas* as predator, the number of individuals on the surface at the beginning of the runs in the *Salinity choice experiment* was clearly larger for stressed *C. edule*, most of which were completely out of the sediment, followed by some stressed individuals of *R. philippinarum* and very few of *V. corrugata* (Fig. 7a,b). At the end of the runs, the number of *C. edule* remaining on surface decreased, particularly in S5 followed by S10 (Fig. 7c), and most of those in S35 were buried (Fig. 7d). Individuals of *V. corrugata* were almost completely buried independently of the treatment both initially and at termination. Few individuals of *R. philippinarum* were buried under increasing stress compared to those buried in the controls (Fig. 7d). In the *Prey choice experiment* the pattern was similar, although very few individuals were completely exposed (Fig. 7e) and most stressed *C. edule* and *R. philippinarum* were at least semi-buried at the beginning of the experiment (Fig. 7f). At the end of the experiment, the number of individuals found on the surface increased slightly for all species and treatments, except perhaps *C. edule* in S10 (Fig. 7g). Overall, the number of buried individuals showed no pattern for any species (Fig. 7h).

**Fig. 8:**
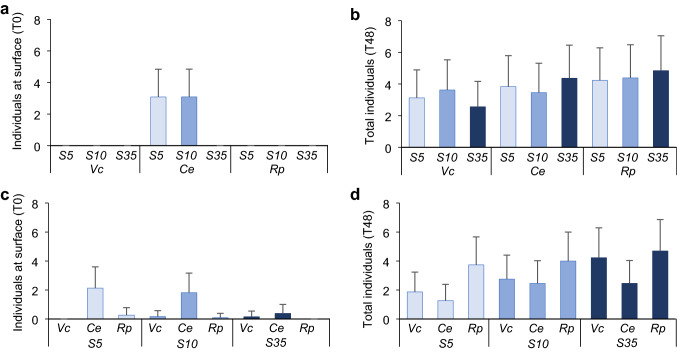
Results of the experiments with *B.brandaris.* Mean (+ SD, *n* = 16–45) of **a** number of individual bivalves found at surface at the beginning of the experiment (T0) and **b** total number of individuals found at the end of the experiment (T48) by treatment (S5, S10, S35) and prey species (*Vc*: *V. corrugata*, *Ce*: *C. edule, Rp*: *R. philippinarum*) in the *Salinity choice experiment,* **c** number of individual bivalves found at surface at the beginning of the experiment (T0) and **d** total number of individuals found at the end of the experiment (T48) by treatment (S5, S10, S35) and prey species (*Vc*: *V. corrugata*, *Ce*: *C. edule, Rp*: *R. philippinarum*) in the *Prey choice experiment*

When using *B. brandaris* as predator, the only species found on the sediment surface in the *Salinity choice experiment* was *C. edule*, particularly when individuals were stressed (Fig. 8a). This was again the species with most of individuals on surface at initiation in the *Prey choice experiment* (Fig. 8c). Most of the remaining bivalves at the end of both experiments were found buried (Fig. 8b, d).

## Discussion

Understanding the consequences of current and projected salinity fluctuations on predator–prey interactions in highly managed systems, such as intertidal shellfish beds, is relevant, because they influence abundance and distribution patterns and population dynamics of species within the ecosystem (Sánchez-Salazar et al. 1987). Although an increase of extreme events can also negatively affect predator dynamics through processes like recruitment (Seed 1993), scope for growth (Stickle and Bayne 1987) and juvenile survival (Covernton and Harley 2020), salinity decreases may be even more unfavourable for less mobile species, such as bivalves (Domínguez et al. 2020).

Stressed prey are likely more vulnerable to predation, because their response to attack may be hindered, and/or their ability to find a refuge can be reduced as a consequence of stress (Tallqvist 2001). As initially predicted, our results showed that vulnerability of stressed prey to predators depended on the prey species. This was especially true with the shore crab *C. maenas*, one of the most important predators of many bivalve species (Masski and Guillou 1999; Curtis et al. 2012). With *C. edule* and *V. corrugata* both stress treatments (S5 and S10) were below the physiological salinity threshold of 15 previously reported for these species (Domínguez et al. 2020), while for *R. philippinarum* differences in vulnerability were only evident for the lowest salinity treatment (S5) perhaps due to its larger tolerance to salinity stress (Domínguez et al. 2020). Such vulnerability of prey was reflected in the total consumption by crabs in 24 h, which was greater on stressed prey. Additionally, non-stressed prey had longer handling times (HBT) and fewer were eaten in the initial experimental period (Fig. 4). As intended by the experimental design, the studied bivalves appeared to have reached the threshold below which prey resistance decreased (Witman and Grange 1998; McLeod et al. 2008). Our results suggest that at salinities 5 and 10, these species of bivalves, particularly cockles, were too damaged to recover normal burrowing activity right after the stress (Domínguez et al. 2020), remaining at or near the surface (Online resource 1) (Figs. 7, 8), or failing to respond to predator cues (Beukema and Dekker 2005; Petes et al. 2007; Talmage and Gobler 2011). In the case of *R. philippinarum*, individuals stressed at S10 appeared to be more resistant to predation than at S5, as denoted by a larger number of rejections, while handling time and total and early consumption rates were intermediate between those for individuals in S5 and S35 (Fig. 4). This was not seen in the individuals of *V. corrugata* and *C. edule* in S10 very likely due to the greater resistance of *R. philippinarum* to low salinity (Bidegain and Juanes 2013; Moura et al. 2017; Domínguez et al. 2020).

The handling times of both predators very likely differed depending on the anti-predator traits of bivalves, including shell thickness and valve closure and, burrowing ability (Boulding 1984; Coffen-Smout 1998; Smallegange et al. 2008; Verdelhos et al. 2015; Glaspie et al. 2017). The thickest *R. philippinarum* and the deeper burrower *V. corrugata* were manipulated for longer times before being consumed relative to *C. edule*. The cockle was found more often by the predators at the sediment surface, and has a clear valve gape. Similarly, the salinity stress appeared to cause a reduction in burrowing and valve closure ability, making all species more accessible compared to the S35 treatment. Handling time to the first bite was expected to be shorter for the more stressed prey since evasive prey behaviour to avoid being consumed (e.g., burrowing or valve closure) is often energetically costly (Leonard et al. 1999; Nakoaka 2000) and stressed prey show worse performance (Verdelhos et al. 2015; Domínguez et al. 2020; Woodin et al. 2020). In the *Salinity choice experiment* with *C. maenas,* the most stressed prey of all species had significantly shorter handling times (HBT) as expected (Fig. 4c). Interestingly, the composite metric of handling time and consumption time (HCT) was significantly longer for stressed prey than for non-stressed prey (Fig. 4d). In the *Prey choice experiment* this appeared to be most clear in consumption of *V. corrugata*, which is known to produce more degradation compounds than *R. philippinarum* at low salinities (Carregosa et al. 2014). If prey selection follows the energy maximization premise (MacArthur and Pianka 1966), our results suggest that crabs maximized energy by consuming more stressed prey, but at the expense of increased time expenditure in consumption. This result apparently violates Charnov’s optimization theory (Charnov 1976), as crabs spent too much time handling each individual prey for the energy gain they might represent. The degradation of flesh in stressed individuals might make difficult the total consumption of the bivalve in few movements so the crabs spent more time to finish the consumption. Behaviour of crabs is very plastic and, in absence of predators, they select smaller prey, therefore decreasing handling time (Smallegange and van der Meer 2003) to minimise potential chelae damage (Smallegange et al. 2008) as crabs very often break part of their dactylus, and in some instances lose their chelipeds when attempting to crush clams (Juanes and Hartwick 1990). This risk could be also minimised by feeding on more vulnerable prey that offer less resistance, maybe because of gaping that facilitated the access to prey after stress. In this study, most of the shells preyed upon by crabs were intact, apart from few individuals, mainly *V. corrugata* and *C. edule,* which were found broken by chipping (Online resource 2).

The response of the gastropod *B. brandaris* differed from that of the shore crabs, because the gastropod consumed prey irrespectively of salinity exposure, except for *C. edule*. Gastropods may be less selective in their prey choice due to lower mobility (Vasconcelos et al. 2008), and predate upon prey that are easier to catch and open. They typically open bivalve shells by drilling or marginal chipping, depending on the prey size (Peharda and Morton 2006) as confirmed in our experiments in which all empty shells preyed by gastropods were intact and showed no evidence of drilling or damage.

When prey species were offered together, the overall total consumption of *C. maenas* was greater on stressed *V. corrugata* in S10, and over both *V. corrugata* and *C. edule* in S5, although this difference was not significant. This contrasted with the previous experiment offering prey species separately, with a greater total consumption of *C. edule*, as expected given the great consumption of crabs of cockles in the field (Sánchez-Salazar et al. 1987; Whitton et al. 2012). This response can be the result of a combination of morphological (i.e. size, shape and shell thickness) and behavioural features of prey (Flynn and Smee 2010; Campbell et al. 2019). *Venerupis corrugata* appear to possess a weaker and thinner shell compared to the other two species which are known to resist cracking (Coffen-Smout 1998; Brom and Szopa 2016) and juveniles used in the experiment were thinner (personal observation). This characteristic, together with the fact that *V. corrugata* is quite vulnerable to low salinity (Domínguez et al. 2020), might result in a greater consumption of crabs upon this species despite that *C. edule* showed similar vulnerability to low salinity (Domínguez et al. 2020). In contrast, *R. philippinarum* is a species more able to resist predation by its shell thickness and valve closure behaviour (Domínguez et al. 2020).

Burrowing behaviour, which is energetically costly, can be compromised by salinities ≤ 15 (Domínguez et al. 2020; Woodin et al. 2020), and could also contribute to the observed patterns of consumption by crabs. This was reflected in the control treatment (S35), in which non-stressed clams and cockles were less consumed. If physiological and behavioural conditions are not compromised by stress, moving deeper into the sediment provides bivalves with spatial refuge from crabs and other predators. In the case of *V. corrugata*, this species often buries deeper than the others (~ 7 cm) (Macho et al. 2006), and even if not found on the surface as the cockles, their ability may be compromised (Domínguez et al. 2020) and, thus, may be found shallower. This, along with chemical signals released by damaged individuals may attract predators (Hayden et al. 2007; Hay 2009; Zimmer-Faust et al. 1995). The gastropod clearly consumed more cockles over the other two bivalve species, independently of the treatments, and this difference increased when non-stressed individuals were offered (Fig. 6). This result reinforces the preference for this prey species that was more often found on the surface, which facilitated their capture, compared to the other two clams (Verdelhos et al. 2015; Domínguez et al. 2020).

In summary, stressed *V. corrugata* and *C. edule* were more vulnerable, although to a lesser extent compared to what is described in most studies in the literature. They were often conducted without sediment, the burrowing refuge for the bivalves and, therefore, found much greater predation rates (Mascaró and Seed 2001; Breen and Metaxas 2008; Curtis et al. 2012). The two predators showed different foraging behaviour because *C. maenas* showed a greater consumption over *V. corrugata* and *C. edule,* whereas *B. brandaris* consistently consumed more *C. edule* in the choice experiments. This might indicate a greater profitability of each prey species for each predator related to a greater availability for capture and consumption (Seed 1993) and/or the ratio of energy content to handling time for these two prey species (Charnov 1976). Feeding behaviour of predators changed with the level of salinity stress of prey: the more sensitive and mobile *C. maenas* detected rapidly more vulnerable, stressed prey likely through chemical cues (Hayden et al. 2007; Hay 2009), while *B. brandaris* showed lower selection ability, likely related to its lower mobility despite using chemical cues to find prey (Croll 1983).

Galician shellfish beds are currently affected by heavy rain events (Parada et al. 2012; Domínguez et al. 2020) that are predicted to increase in frequency and intensity in the short-term future at least in the winter season (Jacob et al. 2014; Cardoso Pereira et al. 2020; Lorenzo and Alvarez 2020). Such changes may cause considerable impacts on structure and dynamics of populations of commercially important bivalves either directly and/or indirectly via alteration of predator–prey interactions. This information is particularly useful for shellfish stakeholders, by detailing risks for commercially important species from the native *C. maenas* or other invasive predators that are becoming abundant on shellfish beds.

## Supplementary Information

Below is the link to the electronic supplementary material.

Supplementary file1 (DOCX 197 kb)

Supplementary file2 (DOCX 10035 kb)

Supplementary file3 (DOCX 29 kb)

Supplementary file4 (DOCX 31 kb)
