# Supplementary material for: Predation risk increases in estuarine bivalves stressed by low salinity
Source: Mar Biol. 2021 Jul 24;168(8):132. doi: 10.1007/s00227-021-03942-8 (PMC8550793; doi:10.1007/s00227-021-03942-8)
Supplement: Supplementary file 1 — Supplementary file1 (DOCX 197 kb) [file 227_2021_3942_MOESM1_ESM.docx]

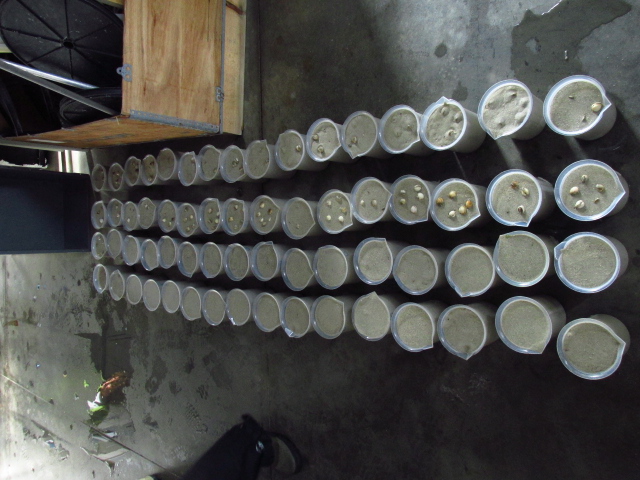


Experimental containers with sediment and 5 individuals of *C. edule* after salinity treatments. From left to right: procedural control without bivalves, S35, S5, S10.
