# Supplementary material for: Predation risk increases in estuarine bivalves stressed by low salinity
Source: Mar Biol. 2021 Jul 24;168(8):132. doi: 10.1007/s00227-021-03942-8 (PMC8550793; doi:10.1007/s00227-021-03942-8)
Supplement: Supplementary file 2 — Supplementary file2 (DOCX 10035 kb) [file 227_2021_3942_MOESM2_ESM.docx]

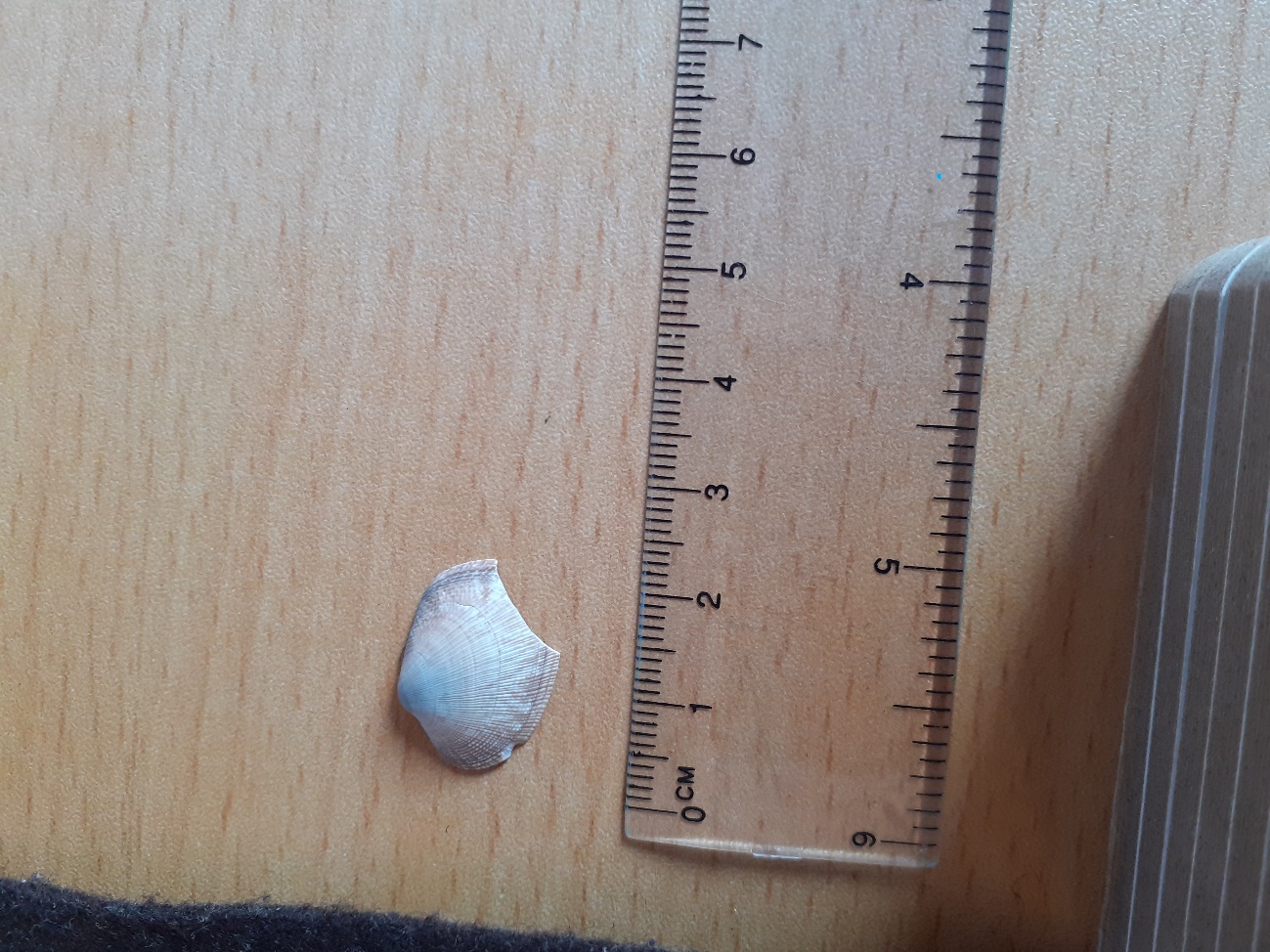

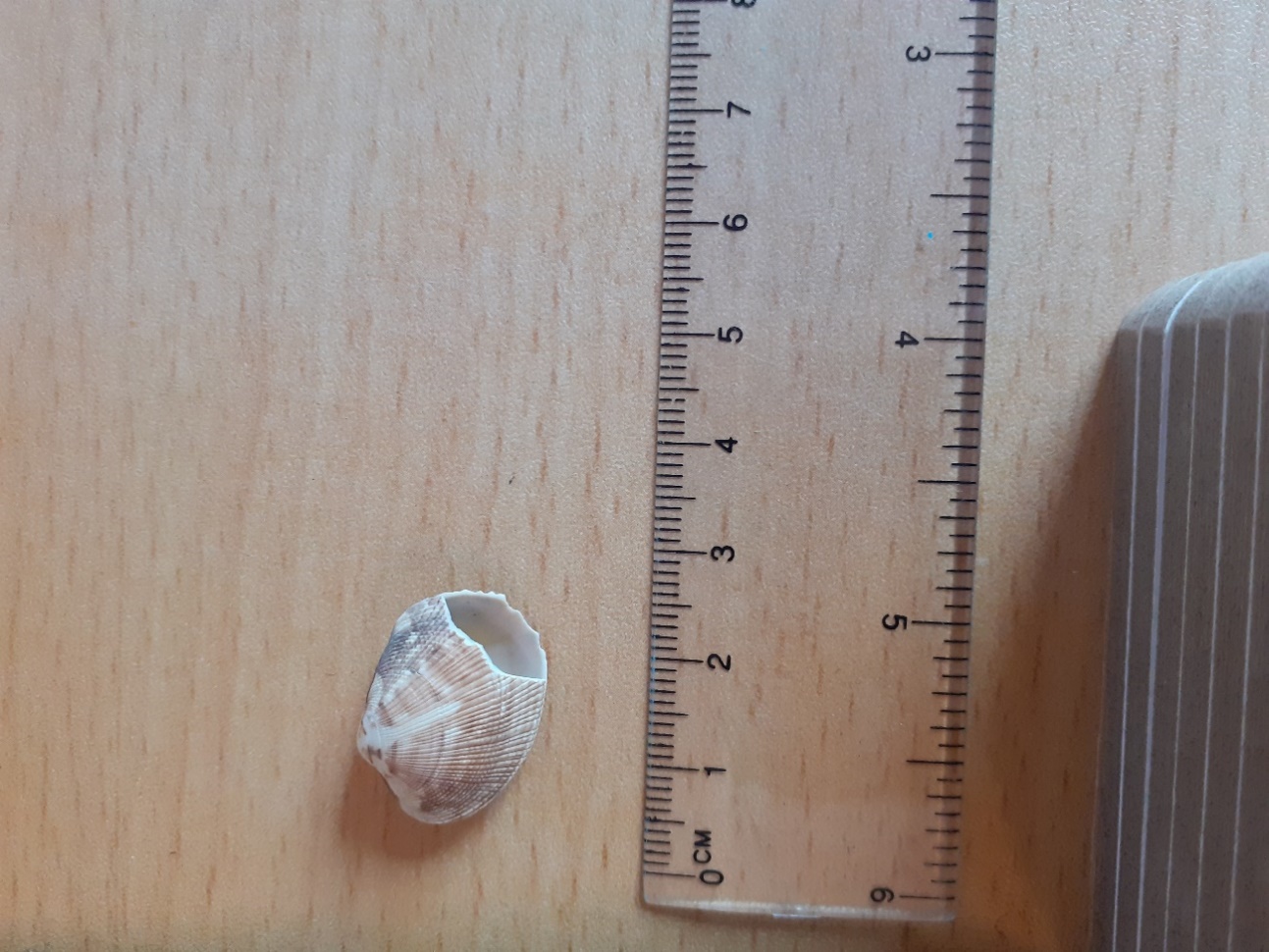

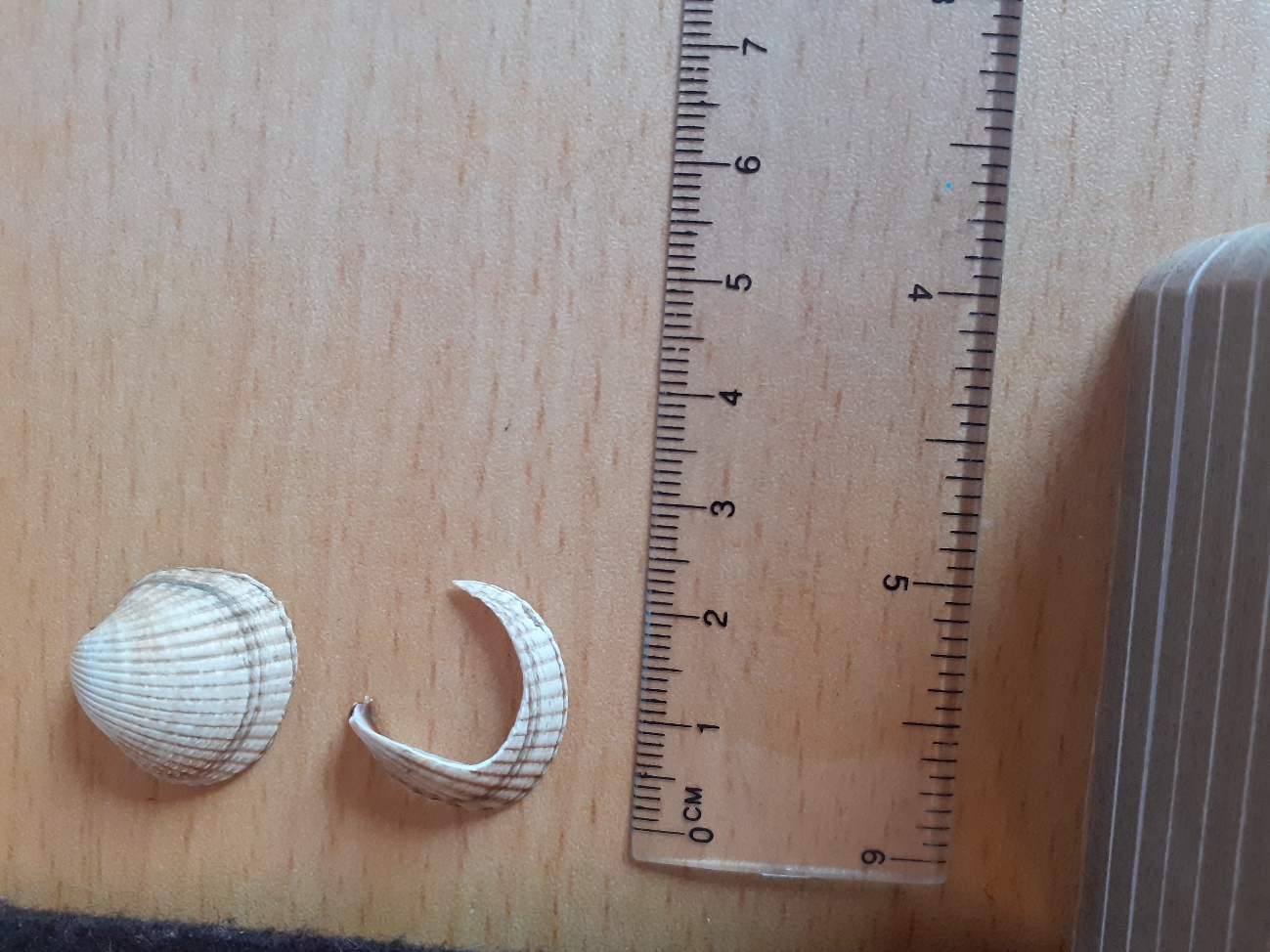

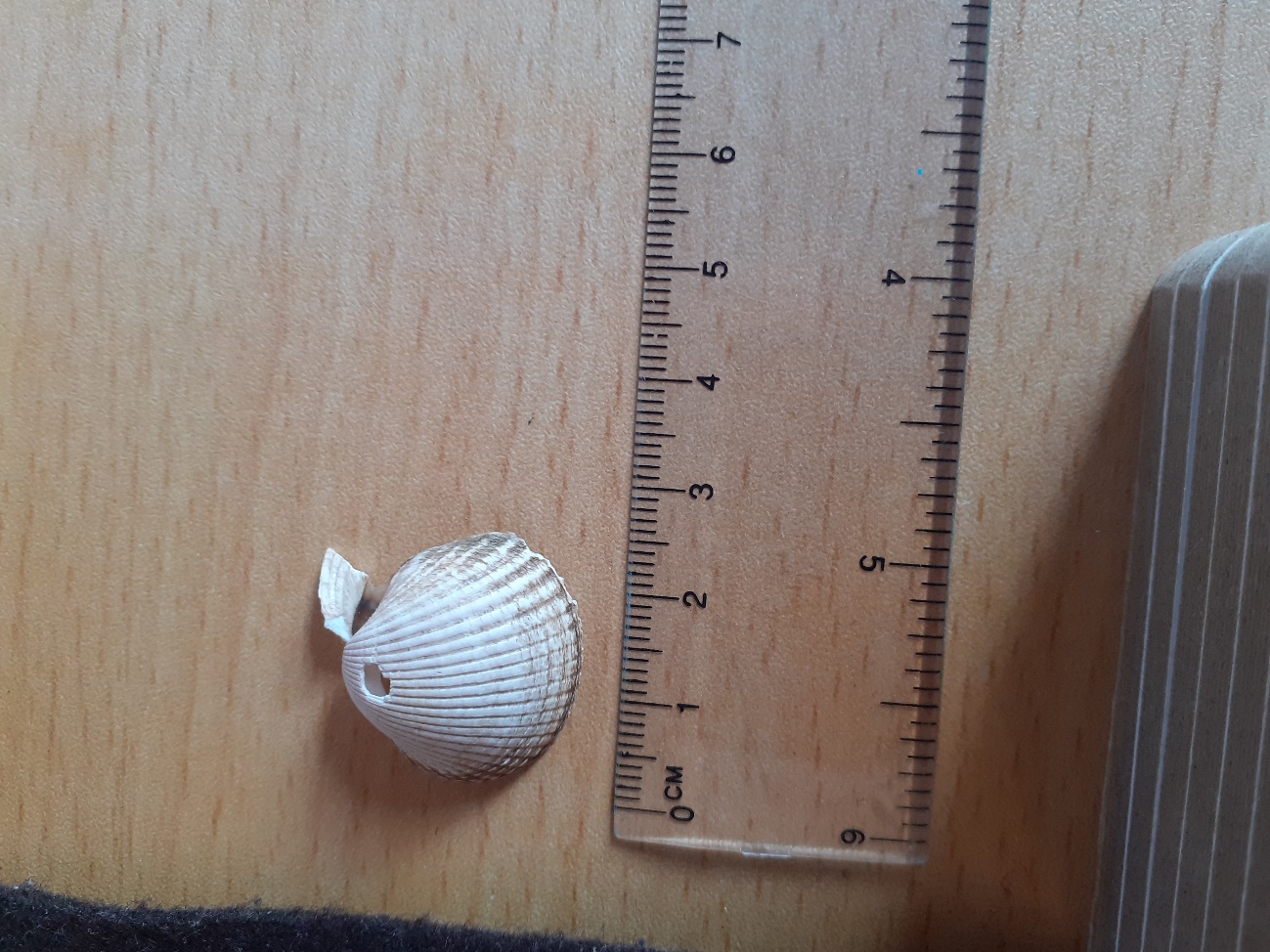

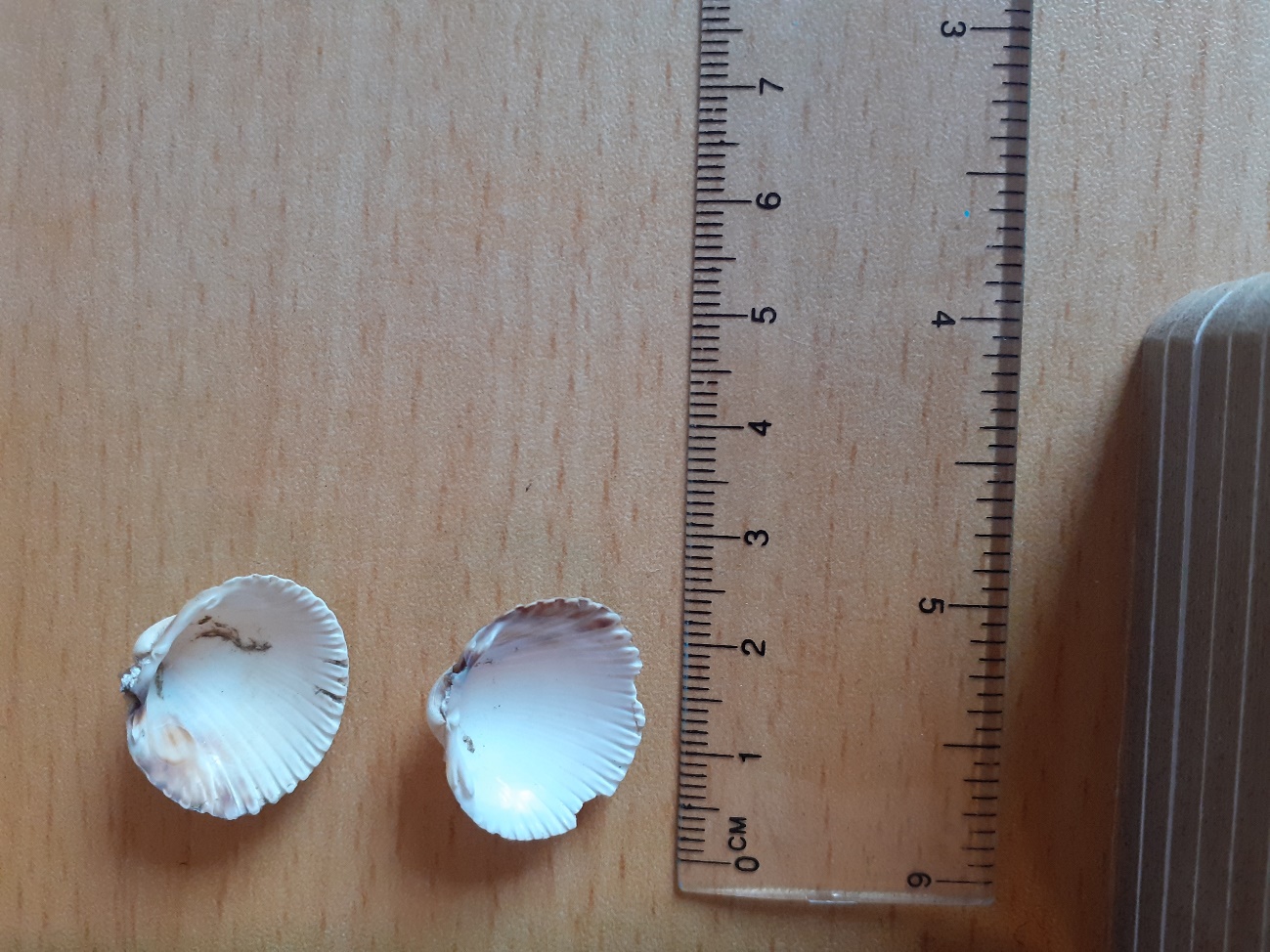

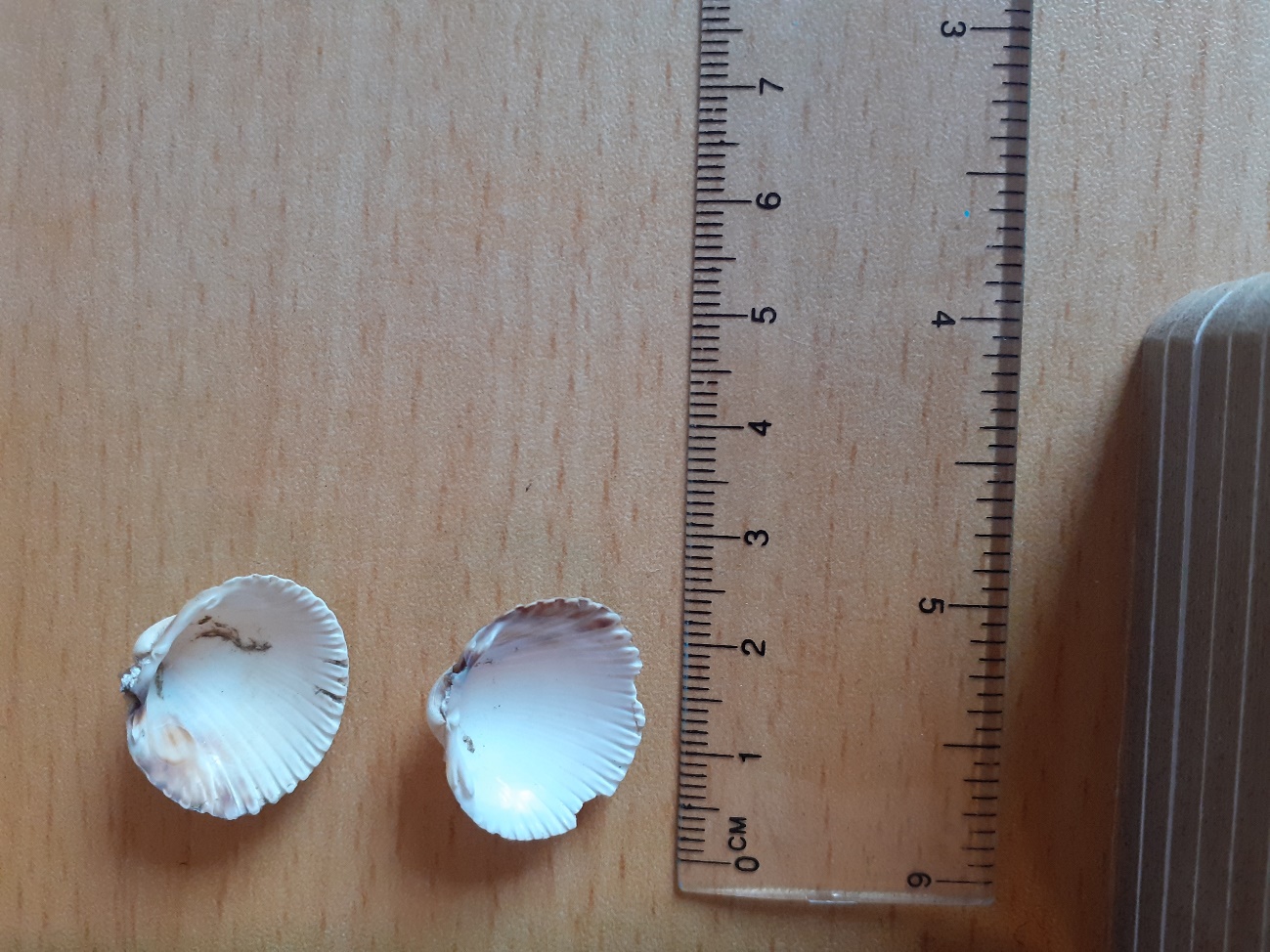

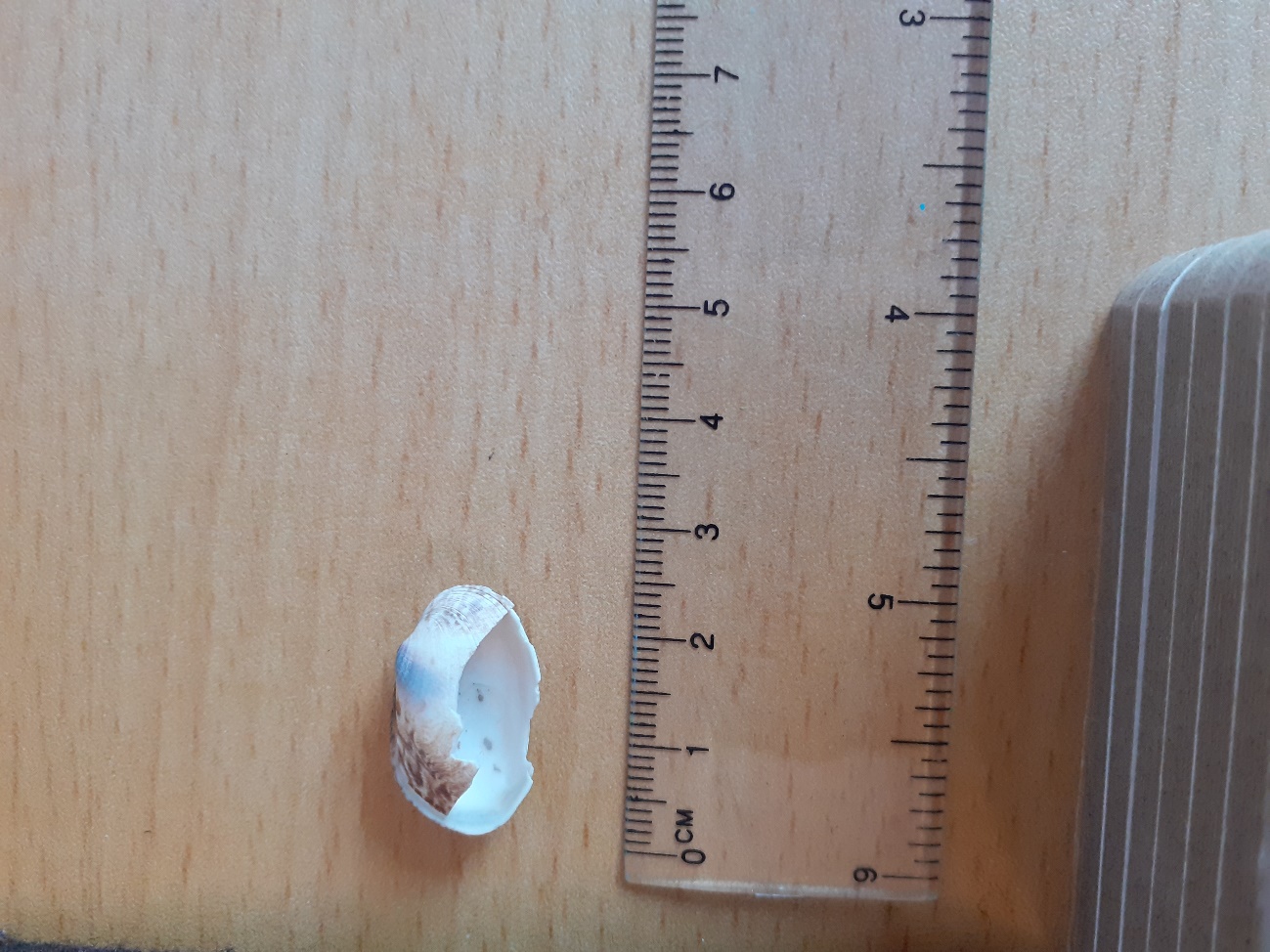

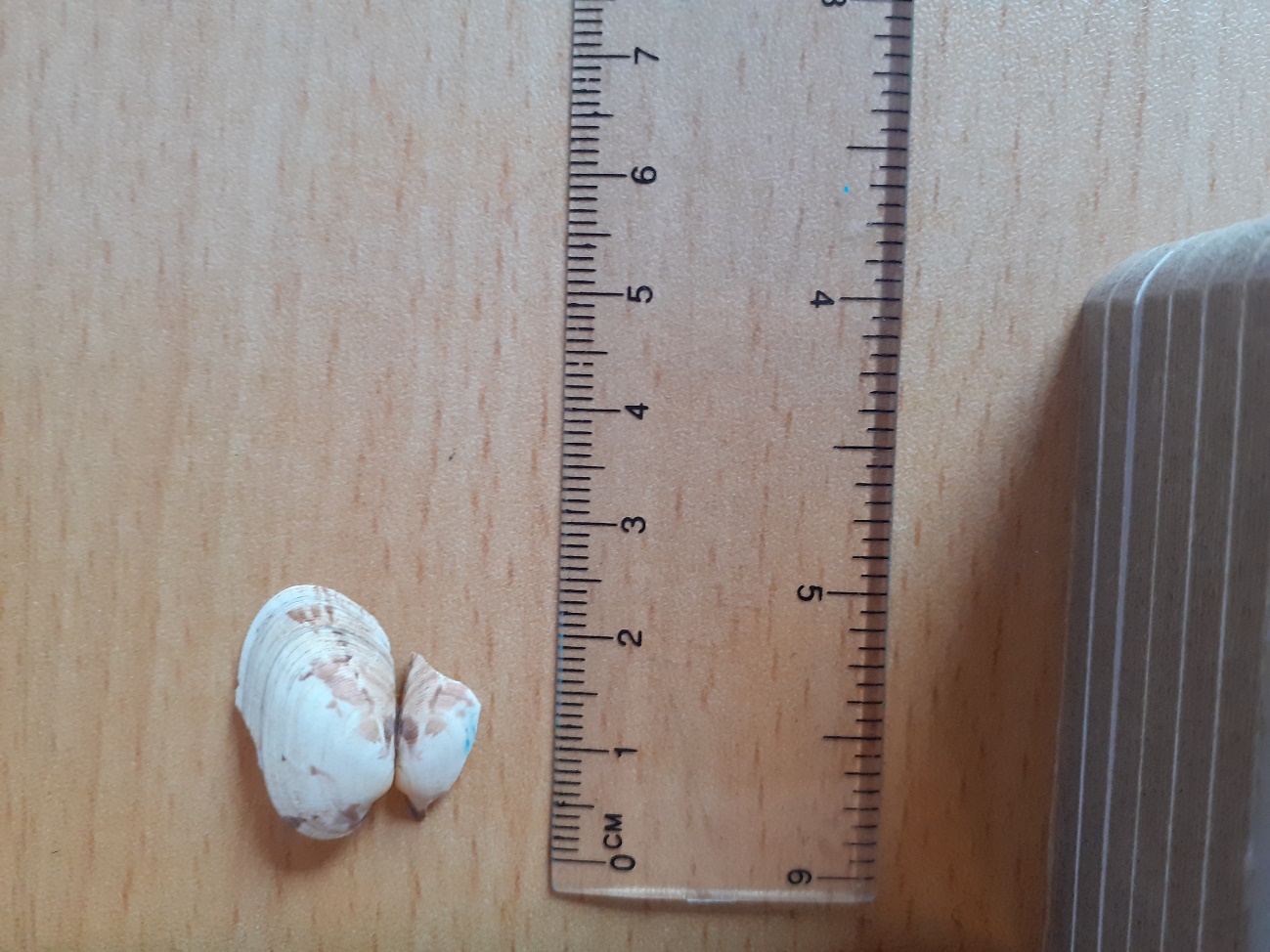

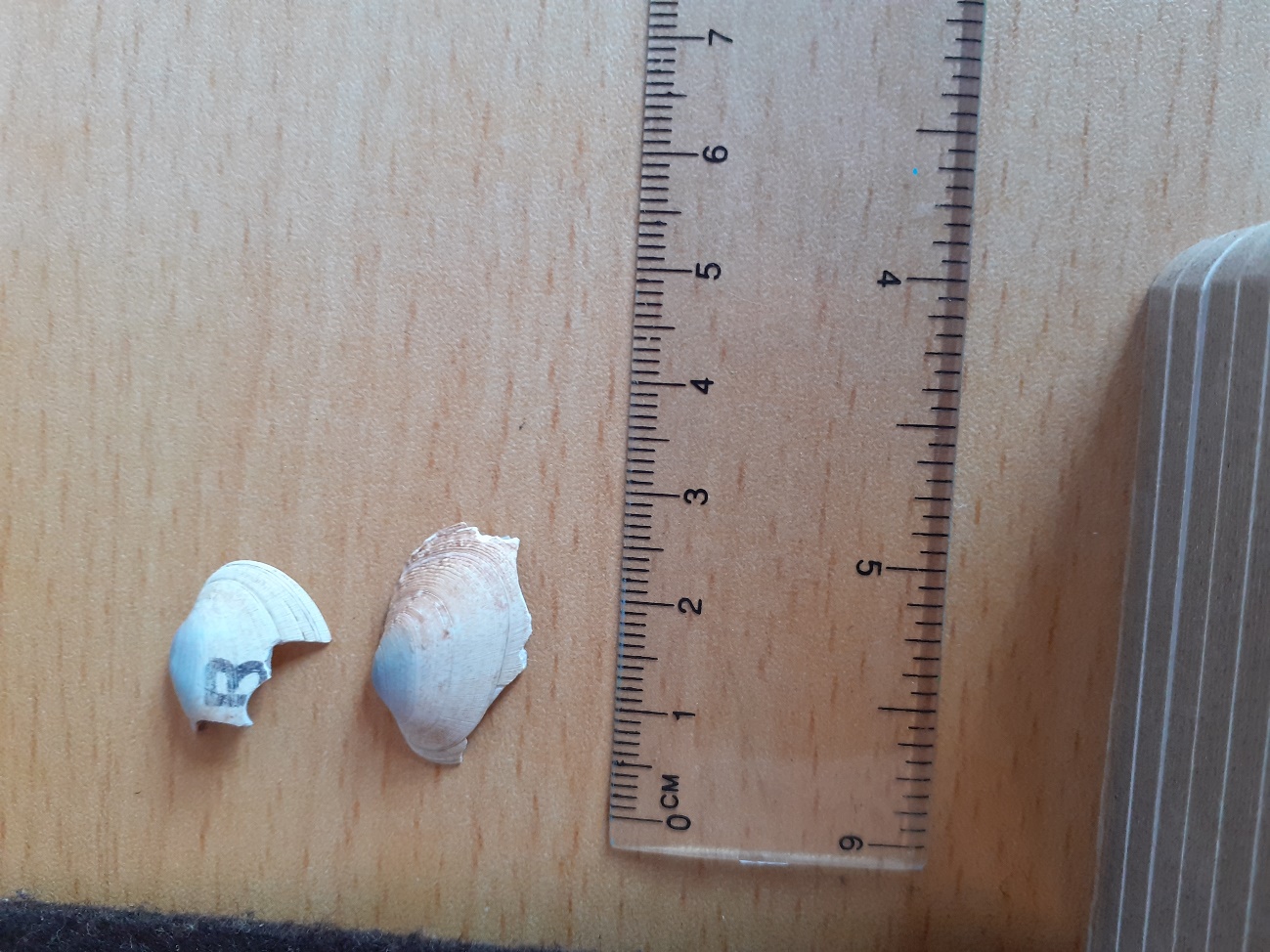


*R. philippinarum*

*C. edule*

*V. corrugata*

Figure S2. Shell remains of the three bivalve species eaten by *C. maenas*.
