# Supplementary material for: Predation risk increases in estuarine bivalves stressed by low salinity
Source: Mar Biol. 2021 Jul 24;168(8):132. doi: 10.1007/s00227-021-03942-8 (PMC8550793; doi:10.1007/s00227-021-03942-8)
Supplement: Supplementary file 3 — Supplementary file3 (DOCX 29 kb) [file 227_2021_3942_MOESM3_ESM.docx]

Table S1. Summary of GLMMs and LMM testing the effect of salinity treatments on prey consumption, HBT and HCT (handling time to first bite and handling time plus period of consumption, respectively) rejections and fraction consumed upon capture in the first 4 hours for the *Salinity choice experiment* with *C. maenas.* The model used Salinity treatment (Treat) as fixed factor, Aquarium as random factor and predator size as covariate. Post-hoc tests results for Salinity treatment (S5, S10, S35) are shown. * Data were arcsine-square root transformed.

| Variable | Prey species | Replicates (Aquarium) | Full model | Parameter | χ² | Df | p | Variance | SD | Post-hoc | P |
| --- | --- | --- | --- | --- | --- | --- | --- | --- | --- | --- | --- |
| Early consumption | *V. corrugata* | n= 8 | Cons ~ Treat + Size  + (1 \| Aquarium) | Random effect |  |  |  |  |  |  |  |
|  |  |  |  | *Aquarium* |  |  |  | 0 | 0 |  |  |
|  |  |  |  | Fixed effects |  |  |  |  |  |  |  |
|  |  |  |  | *Intercept* | 3.24 | 1 | 0.072 |  |  |  |  |
|  |  |  |  | *Treat* | 4.11 | 2 | 0.128 |  |  |  |  |
|  |  |  |  | *Size* | 1.72 | 1 | 0.189 |  |  |  |  |
|  | *C. edule* | n= 11 | Cons ~ Treat + Size  + (1 \| Aquarium) | Random effect |  |  |  |  |  |  |  |
|  |  |  |  | *Aquarium* |  |  |  | 0 | 0 |  |  |
|  |  |  |  | Fixed effects |  |  |  |  |  |  |  |
|  |  |  |  | *Intercept* | 14.14 | 1 | **<0.001** |  |  |  |  |
|  |  |  |  | *Treat* | 11.44 | 2 | **0.003** |  |  | *S5- S35* | **<0.01** |
|  |  |  |  | *Size* | 0.77 | 1 | 0.38 |  |  | *S10- S35* | **<0.01** |
|  | *R. philippinarum* | n= 13 | Cons ~ Treat + Size  + (1 \| Aquarium) | Random effect |  |  |  |  |  |  |  |
|  |  |  |  | *Aquarium* |  |  |  | 0 | 0 |  |  |
|  |  |  |  | Fixed effects |  |  |  |  |  |  |  |
|  |  |  |  | *Intercept* | 1.69 | 1 | 0.194 |  |  |  |  |
|  |  |  |  | *Treat* | 9.01 | 2 | **0.011** |  |  | *S5- S35* | **0.011** |
|  |  |  |  | *Size* | 0.93 | 1 | 0.334 |  |  |  |  |
| HBT | *V. corrugata* | n= 8 | HT ~ Treat + Size  + (1 \| Aquarium) | Random effect |  |  |  |  |  |  |  |
|  |  |  |  | *Aquarium* |  |  |  | <0.001 | <0.001 |  |  |
|  |  |  |  | Fixed effects |  |  |  |  |  |  |  |
|  |  |  |  | *Intercept* | 0.79 | 1 | 0.37 |  |  |  |  |
|  |  |  |  | *Treat* | 2.28 | 2 | **0.04** |  |  |  |  |
|  |  |  |  | *Size* | 0.98 | 1 | 0.32 |  |  | *S10- S35* | **0.03** |
|  | *C. edule* | n= 11 | HT ~ Treat + Size  + (1 \| Aquarium) | Random effect |  |  |  |  |  |  |  |
|  |  |  |  | *Aquarium* |  |  |  | <0.001 | <0.001 |  |  |
|  |  |  |  | Fixed effects |  |  |  |  |  |  |  |
|  |  |  |  | *Intercept* | 0.003 | 1 | 0.95 |  |  |  |  |
|  |  |  |  | *Treat* | 6.12 | 2 | 0.32 |  |  |  |  |
|  |  |  |  | *Size* | 0.41 | 1 | 0.52 |  |  |  |  |
|  | *R. philippinarum* | n= 13 | HT ~ Treat + Size  + (1 \| Aquarium) | Random effect |  |  |  |  |  |  |  |
|  |  |  |  | *Aquarium* |  |  |  | <0.001 | <0.001 |  |  |
|  |  |  |  | Fixed effects |  |  |  |  |  |  |  |
|  |  |  |  | *Intercept* | 17.35 | 1 | **<0.001** |  |  |  |  |
|  |  |  |  | *Treat* | 5.55 | 2 | **0.06** |  |  | *S5- S35* | **0.04** |
|  |  |  |  | *Size* | 0.04 | 1 | 0.84 |  |  |  |  |
| HCT | *V. corrugata* | n= 8 | HT ~ Treat + Size | Random effect |  |  |  |  |  |  |  |
|  |  |  | + (1 \| Aquarium) | *Aquarium* |  |  |  | <0.001 | <0.001 |  |  |
|  |  |  |  | Fixed effects |  |  |  |  |  |  |  |
|  |  |  |  | *Intercept* | 49.64 | 1 | **<0.001** |  |  |  |  |
|  |  |  |  | *Treat* | 9.25 | 2 | **<0.01** |  |  | *S5- S35* | **<0.01** |
|  |  |  |  | *Size* | 0.42 | 1 | 0.51 |  |  | *S10- S35* | **<0.01** |
|  | *C. edule* | n= 11 | HT ~ Treat + Size | Random effect |  |  |  |  |  |  |  |
|  |  |  | + (1 \| Aquarium) | *Aquarium* |  |  |  | <0.001 | <0.001 |  |  |
|  |  |  |  | Fixed effects |  |  |  |  |  |  |  |
|  |  |  |  | *Intercept* | 37.21 | 1 | **<0.001** |  |  |  |  |
|  |  |  |  | *Treat* | 7.85 | 2 | **0.02** |  |  | *S5- S35* | **0.02** |
|  |  |  |  | *Size* | 1.63 | 1 | 0.21 |  |  | *S10- S35* | 0.09 |
|  | *R. philippinarum* | n= 13 | HT ~ Treat + Size | Random effect |  |  |  |  |  |  |  |
|  |  |  | + (1 \| Aquarium) | *Aquarium* |  |  |  | <0.001 | <0.001 |  |  |
|  |  |  |  | Fixed effects |  |  |  |  |  |  |  |
|  |  |  |  | *Intercept* | 63.64 | 1 | **<0.001** |  |  | *S5- S10* | 0.09 |
|  |  |  |  | *Treat* | 17.02 | 2 | **<0.001** |  |  | *S5- S35* | **<0.01** |
|  |  |  |  | *Size* | 0.13 | 1 | 0.72 |  |  |  |  |
| Rejections | *V. corrugata* | n= 8 | Rej ~ Treat + Size | Random effect |  |  |  |  |  |  |  |
|  |  |  | + (1 \| Aquarium) | *Aquarium* |  |  |  | 4.29 | 2.07 |  |  |
|  |  |  |  | Fixed effects |  |  |  |  |  |  |  |
|  |  |  |  | *Intercept* | 0 | 1 | 0.997 |  |  |  |  |
|  |  |  |  | *Treat* | 2.16 | 2 | 0.339 |  |  |  |  |
|  |  |  |  | *Size* | 0.04 | 1 | 0.832 |  |  |  |  |
|  | *C. edule* | n= 11 | Rej ~ Treat + Size | Random effect |  |  |  |  |  |  |  |
|  |  |  | + (1 \| Aquarium) | *Aquarium* |  |  |  | 1.76 | 1.33 |  |  |
|  |  |  |  | Fixed effects |  |  |  |  |  |  |  |
|  |  |  |  | *Intercept* | 5.61 | 1 | **0.018** |  |  |  |  |
|  |  |  |  | *Treat* | 0.56 | 2 | 0.754 |  |  |  |  |
|  |  |  |  | *Size* | 1.51 | 1 | 0.218 |  |  |  |  |
|  | *R. philippinarum* | n= 13 | Rej ~ Treat + Size | Random effect |  |  |  |  |  |  |  |
|  |  |  | + (1 \| Aquarium) | *Aquarium* |  |  |  | 0.71 | 0.84 |  |  |
|  |  |  |  | Fixed effects |  |  |  |  |  |  |  |
|  |  |  |  | *Intercept* | 2.18 | 1 | 0.139 |  |  |  |  |
|  |  |  |  | *Treat* | 15.81 | 2 | **<0.001** |  |  | *S5- S10* | **0.017** |
|  |  |  |  | *Size* | 0.003 | 1 | 0.955 |  |  | *S10- S35* | **<0.01** |
| Fraction consumed * | *V. corrugata* | n= 8 | FC ~ Treat + Size  + (1 \| Aquarium) | Random effect |  |  |  |  |  |  |  |
|  |  |  |  | *Aquarium* |  |  |  | 0.04 | 0.2 |  |  |
|  |  |  |  | Fixed effects |  |  |  |  |  |  |  |
|  |  |  |  | *Intercept* | 3.14 | 1 | **0.076** |  |  |  |  |
|  |  |  |  | *Treat* | 9.92 | 2 | **0.007** |  |  | *S10- S35* | **0.018** |
|  |  |  |  | *Size* | 0.56 | 1 | 0.453 |  |  |  |  |
|  | *C. edule* | n= 11 | FC ~ Treat + Size  + (1 \| Aquarium) | Random effect |  |  |  |  |  |  |  |
|  |  |  |  | *Aquarium* |  |  |  | 0 | 0 |  |  |
|  |  |  |  | Fixed effects |  |  |  |  |  |  |  |
|  |  |  |  | *Intercept* | 40.17 | 1 | **2.3e-10** |  |  |  |  |
|  |  |  |  | *Treat* | 15.55 | 2 | **<0.001** |  |  | *S5- S35* | **<0.01** |
|  |  |  |  | *Size* | 0.11 | 1 | 0.749 |  |  | *S10- S35* | **<0.01** |
|  | *R. philippinarum* | n= 13 | FC ~ Treat + Size  + (1 \| Aquarium) | Random effect |  |  |  |  |  |  |  |
|  |  |  |  | *Aquarium* |  |  |  | 0.006 | 0.08 |  |  |
|  |  |  |  | Fixed effects |  |  |  |  |  |  |  |
|  |  |  |  | *Intercept* | 78.21 | 1 | **<2.2e-16** |  |  |  |  |
|  |  |  |  | *Treat* | 34.32 | 2 | **<0.001** |  |  | *S5- S10* | **<0.001** |
|  |  |  |  | *Size* | 2.61 | 1 | 0.107 |  |  | *S5- S35* | **<0.001** |
