# Supplementary material for: Predation risk increases in estuarine bivalves stressed by low salinity
Source: Mar Biol. 2021 Jul 24;168(8):132. doi: 10.1007/s00227-021-03942-8 (PMC8550793; doi:10.1007/s00227-021-03942-8)
Supplement: Supplementary file 4 — Supplementary file4 (DOCX 31 kb) [file 227_2021_3942_MOESM4_ESM.docx]

Table S2. Summary of GLMMs and LMM testing the effect of salinity treatments on mean prey consumption, rejections, HBT and HCT (handling time to first bite and handling time plus period of consumption, respectively) and fraction consumed upon capture in the first 4 hours for the *Prey choice experiment* with *C. maenas.* The model used Species as fixed factor, Aquarium and Run as random factors and predator size as covariate. Post-hoc tests results for the significant factors are shown. Salinity (S5, S10, S35), Species: Vc: *V. corrugata*, Ce: *C. edule*, Rp; *R. philippinarum*. * Data were arcsine-square-root transformed.

| Variable | Salinity treatment | Replicates (Aquarium) | Full model | Parameter | χ² | Df | p | Variance | SD | Post-hoc | P |
| --- | --- | --- | --- | --- | --- | --- | --- | --- | --- | --- | --- |
| Early consumption | *S5* | n= 30 | Cons ~ Species + Size  + (1 \| Aquarium) + (1 \| Run) | Random effect |  |  |  |  |  |  |  |
|  |  |  |  | *Aquarium* |  |  |  | 0 | 0 |  |  |
|  |  |  |  | *Run* |  |  |  | 0.05 | 0.23 |  |  |
|  |  |  |  | Fixed effects |  |  |  |  |  |  |  |
|  |  |  |  | *Intercept* | 1.59 | 1 | 0.207 |  |  |  |  |
|  |  |  |  | *Species* | 2.11 | 2 | 0.349 |  |  |  |  |
|  |  |  |  | *Size* | 0.01 | 1 | 0.904 |  |  |  |  |
|  | *S10* | n= 36 | Cons ~ Species + Size  + (1 \| Aquarium) + (1 \| Run) | Random effect |  |  |  |  |  |  |  |
|  |  |  |  | *Aquarium* |  |  |  | 0 | 0 |  |  |
|  |  |  |  | *Run* |  |  |  | 0 | 0 |  |  |
|  |  |  |  | Fixed effects |  |  |  |  |  |  |  |
|  |  |  |  | *Intercept* | 0.12 | 1 | 0.734 |  |  |  |  |
|  |  |  |  | *Species* | 7.54 | 2 | **0.023** |  |  | *Vc-Rp* | **0.068** |
|  |  |  |  | *Size* | 0.56 | 1 | 0.455 |  |  |  |  |
|  | *S35* | n= 18 | Cons ~ Species + Size  + (1 \| Aquarium) + (1 \| Run) | Random effect |  |  |  |  |  |  |  |
|  |  |  |  | *Aquarium* |  |  |  | <0.001 | <0.001 |  |  |
|  |  |  |  | *Run* |  |  |  | 0 | 0 |  |  |
|  |  |  |  | Fixed effects |  |  |  |  |  |  |  |
|  |  |  |  | *Intercept* | 0 | 1 | 0.999 |  |  |  |  |
|  |  |  |  | *Species* | 0 | 2 | 1.000 |  |  |  |  |
|  |  |  |  | *Size* | 0.16 | 1 | 0.692 |  |  |  |  |
| HBT | *S5* | n= 30 | HBT ~ Species + Size | Random effect |  |  |  |  |  |  |  |
|  |  |  | + (1 \| Aquarium) + (1 \| Run) | *Aquarium* |  |  |  | 0 | 0 |  |  |
|  |  |  |  | *Run* |  |  |  | 0 | 0 |  |  |
|  |  |  |  | Fixed effects |  |  |  |  |  |  |  |
|  |  |  |  | *Intercept* | 0.45 | 1 | 0.504 |  |  |  |  |
|  |  |  |  | *Species* | 0.66 | 2 | 0.720 |  |  |  |  |
|  |  |  |  | *Size* | 0.04 | 1 | 0.838 |  |  |  |  |
|  | *S10* | n= 36 | HBT ~ Species * Size | Random effect |  |  |  |  |  |  |  |
|  |  |  | + (1 \| Aquarium) + (1 \| Run) | *Aquarium* |  |  |  | <0.001 | <0.001 |  |  |
|  |  |  |  | *Run* |  |  |  | 1.98 | 1.44 |  |  |
|  |  |  |  | Fixed effects |  |  |  |  |  |  |  |
|  |  |  |  | *Intercept* | 0.01 | 1 | 0.906 |  |  |  |  |
|  |  |  |  | *Species* | 6.25 | 2 | **0.044** |  |  | *-* | - |
|  |  |  |  | *Size* | 0.32 | 1 | 0.104 |  |  |  |  |
|  |  |  |  | *Species*Size* | 4.53 | 2 | 0.104 |  |  |  |  |
|  | *S35* | n= 18 | HT ~ Species + Size | Random effect |  |  |  |  |  |  |  |
|  |  |  | + (1 \| Aquarium) + (1 \| Run) | *Aquarium* |  |  |  | 0.24 | 0.49 |  |  |
|  |  |  |  | *Run* |  |  |  | 0 | 0 |  |  |
|  |  |  |  | Fixed effects |  |  |  |  |  |  |  |
|  |  |  |  | *Intercept* | 0.004 | 1 | 0.95 |  |  |  |  |
|  |  |  |  | *Species* | 2.15 | 2 | 0.34 |  |  |  |  |
|  |  |  |  | *Size* | 0.13 | 1 | 0.71 |  |  |  |  |
| HCT | *S5* | n= 30 | HT ~ Species + Size | Random effect |  |  |  |  |  |  |  |
|  |  |  | + (1 \| Aquarium) + (1 \| Run) | *Aquarium* |  |  |  | <0.001 | <0.001 |  |  |
|  |  |  |  | *Run* |  |  |  | 0 | 0 |  |  |
|  |  |  |  | Fixed effects |  |  |  |  |  |  |  |
|  |  |  |  | *Intercept* | 7.72 | 1 | **<0.01** |  |  |  |  |
|  |  |  |  | *Species* | 2.09 | 2 | 0.35 |  |  |  |  |
|  |  |  |  | *Size* | 0.17 | 1 | 0.68 |  |  |  |  |
|  | *S10* | n= 36 | HT ~ Species + Size | Random effect |  |  |  |  |  |  |  |
|  |  |  | + (1 \| Aquarium) + (1 \| Run) | *Aquarium* |  |  |  | <0.001 | <0.001 |  |  |
|  |  |  |  | *Run* |  |  |  | <0.001 | <0.001 |  |  |
|  |  |  |  | Fixed effects |  |  |  |  |  |  |  |
|  |  |  |  | *Intercept* | 7.21 | 1 | **<0.01** |  |  |  |  |
|  |  |  |  | *Species* | 3.78 | 2 | 0.15 |  |  |  |  |
|  |  |  |  | *Size* | 1.26 | 1 | 0.53 |  |  |  |  |
|  | *S35* | n= 18 | HT ~ Species + Size | Random effect |  |  |  |  |  |  |  |
|  |  |  | + (1 \| Aquarium) + (1 \| Run) | *Aquarium* |  |  |  | 9.05 | 9.51 |  |  |
|  |  |  |  | *Run* |  |  |  | <0.01 | <0.01 |  |  |
|  |  |  |  | Fixed effects |  |  |  |  |  |  |  |
|  |  |  |  | *Intercept* | 0 | 1 | 0.999 |  |  |  |  |
|  |  |  |  | *Species* | <0.01 | 2 | 0.999 |  |  |  |  |
|  |  |  |  | *Size* | <0.01 | 1 | 0.978 |  |  |  |  |
| Rejection | *S5* | n= 30 | Rej ~ Species + Size  + (1 \| Aquarium) + (1 \| Run) | Random effect |  |  |  |  |  |  |  |
|  |  |  |  | *Aquarium* |  |  |  | 2.29 | 1.52 |  |  |
|  |  |  |  | *Run* |  |  |  | 1.79 | 1.34 |  |  |
|  |  |  |  | Fixed effects |  |  |  |  |  |  |  |
|  |  |  |  | *Intercept* | 0 | 1 | 0.99 |  |  |  |  |
|  |  |  |  | *Species* | 0 | 2 | 1.00 |  |  |  |  |
|  |  |  |  | *Size* | 0.67 | 1 | 0.41 |  |  |  |  |
|  | *S10* | n= 36 | Rej ~ Species + Size  + (1 \| Aquarium) + (1 \| Run) | Random effect |  |  |  |  |  |  |  |
|  |  |  |  | *Aquarium* |  |  |  | 0.65 | 0.81 |  |  |
|  |  |  |  | *Run* |  |  |  | <0.001 | <0.001 |  |  |
|  |  |  |  | Fixed effects |  |  |  |  |  |  |  |
|  |  |  |  | *Intercept* | 0 | 1 | 0.99 |  |  |  |  |
|  |  |  |  | *Species* | 2.06 | 2 | 0.36 |  |  |  |  |
|  |  |  |  | *Size* | 0.08 | 1 | 0.77 |  |  |  |  |
|  | *S35* | n= 18 | Rej ~ Species + Size  + (1 \| Aquarium) + (1 \| Run) | Random effect |  |  |  |  |  |  |  |
|  |  |  |  | *Aquarium* |  |  |  | 2.23 | 1.52 |  |  |
|  |  |  |  | *Run* |  |  |  | 2.56 | 1.59 |  |  |
|  |  |  |  | Fixed effects |  |  |  |  |  |  |  |
|  |  |  |  | *Intercept* | 2.33 | 1 | 0.127 |  |  |  |  |
|  |  |  |  | *Species* | 7.71 | 2 | **0.021** |  |  | *Vc- Ce* | **0.01** |
|  |  |  |  | *Size* | 5.21 | 1 | **0.022** |  |  | *Vc- Rp* | **0.04** |
| Fraction consumed * | *S5* | n= 30 | FC ~ Species + Size  + (1 \| Aquarium) + (1 \| Run) | Random effect |  |  |  |  |  |  |  |
|  |  |  |  | *Aquarium* |  |  |  | 0 | 0 |  |  |
|  |  |  |  | *Run* |  |  |  | 0 | 0 |  |  |
|  |  |  |  | Fixed effects |  |  |  |  |  |  |  |
|  |  |  |  | *Intercept* | 4.12 | 1 | **0.042** |  |  |  |  |
|  |  |  |  | *Species* | 0.53 | 2 | 0.766 |  |  |  |  |
|  |  |  |  | *Size* | 0.18 | 1 | 0.664 |  |  |  |  |
|  | *S10* | n= 36 | FC ~ Species + Size  + (1 \| Aquarium) + (1 \| Run) | Random effect |  |  |  |  |  |  |  |
|  |  |  |  | *Aquarium* |  |  |  | <0.001 | <0.001 |  |  |
|  |  |  |  | *Run* |  |  |  | 0.03 | 0.02 |  |  |
|  |  |  |  | Fixed effects |  |  |  |  |  |  |  |
|  |  |  |  | *Intercept* | 10.27 | 1 | **0.001** |  |  |  |  |
|  |  |  |  | *Species* | 5.64 | 2 | **0.059** |  |  | *Vc- Rp* | **0.073** |
|  |  |  |  | *Size* | 0.17 | 1 | 0.682 |  |  |  |  |
|  | *S35* | n= 18 | FC ~ Species + Size  + (1 \| Aquarium) + (1 \| Run) | Random effect |  |  |  |  |  |  |  |
|  |  |  |  | *Aquarium* |  |  |  | <0.001 | <0.01 |  |  |
|  |  |  |  | *Run* |  |  |  | 0 | 0 |  |  |
|  |  |  |  | Fixed effects |  |  |  |  |  |  |  |
|  |  |  |  | *Intercept* | 0.004 | 1 | 0.951 |  |  |  |  |
|  |  |  |  | *Species* | 2.15 | 2 | 0.341 |  |  |  |  |
|  |  |  |  | *Size* | 0.13 | 1 | 0.713 |  |  |  |  |
